# Evaluating Evidence-Based Content, Features of Exercise Instruction, and Expert Involvement in Physical Activity Apps for Pregnant Women: Systematic Search and Content Analysis

**DOI:** 10.2196/31607

**Published:** 2022-01-19

**Authors:** Melanie J Hayman, Kristie-Lee Alfrey, Kim Waters, Summer Cannon, Gregore I Mielke, Shelley E Keating, Gabriela P Mena, Michelle F Mottola, Kelly R Evenson, Margie H Davenport, S Ariel Barlow, Emily Budzynski-Seymour, Natalie Comardelle, Madison Dickey, Cheryce L Harrison, Maryam Kebbe, Trine Moholdt, Lisa J Moran, Taniya S Nagpal, Stephanie Schoeppe, Stephanie Alley, Wendy J Brown, Susan Williams, Lisa Vincze

**Affiliations:** 1 Appleton Institute School of Health, Medical and Applied Sciences Central Queensland University Rockhampton Australia; 2 Centre for Research on Exercise, Physical Activity and Health School of Human Movement and Nutrition Sciences The University of Queensland Brisbane Australia; 3 School of Human Movement and Nutrition Sciences The University of Queensland Brisbane Australia; 4 R Samuel McLaughlin Foundation Exercise and Pregnancy Laboratory, School of Kinesiology University of Western Ontario London, ON Canada; 5 Department of Anatomy & Cell Biology University of Western Ontario London, ON Canada; 6 Children’s Health Research Institute University of Western Ontario London, ON Canada; 7 Department of Epidemiology University of North Carolina Chapel Hill, NC United States; 8 Faculty of Kinesiology, Sport, and Recreation University of Alberta Edmonton, AB Canada; 9 Reproductive Endocrinology & Women’s Health Laboratory Pennington Biomedical Research Center Louisiana State University Baton Rouge, LA United States; 10 Faculty of Sport, Health and Social Sciences Solent University Southampton United Kingdom; 11 Monash Centre for Health Research and Implementation School of Public Health and Preventive Medicine Monash University Melbourne Australia; 12 Department of Circulation and Medical Imaging Norwegian University of Science and Technology Trondheim Norway; 13 Women’s Clinic St. Olavs University Hospital Trondheim Norway; 14 Faculty of Applied Health Sciences School of Kinesiology Brock University Niagara Region, ON Canada; 15 School of Health Sciences and Social Work Griffith University Gold Coast Australia; 16 Menzies Health Institute Queensland Griffith Health Centre Gold Coast Australia

**Keywords:** apps, exercise, mobile health, mHealth, mobile phone, physical activity, pregnancy, exercise prescription, evidence-based guidelines, app development, systematic review, mobile phone

## Abstract

**Background:**

Guidelines for physical activity and exercise during pregnancy recommend that all women without contraindications engage in regular physical activity to improve both their own health and the health of their baby. Many women are uncertain how to safely engage in physical activity and exercise during this life stage and are increasingly using mobile apps to access health-related information. However, the extent to which apps that provide physical activity and exercise advice align with current evidence-based pregnancy recommendations is unclear.

**Objective:**

This study aims to conduct a systematic search and content analysis of apps that promote physical activity and exercise in pregnancy to examine the alignment of the content with current evidence-based recommendations; delivery, format, and features of physical activity and exercise instruction; and credentials of the app developers.

**Methods:**

Systematic searches were conducted in the Australian App Store and Google Play Store in October 2020. Apps were identified using combinations of search terms relevant to pregnancy and exercise or physical activity and screened for inclusion (with a primary focus on physical activity and exercise during pregnancy, free to download or did not require immediate paid subscription, and an average user rating of ≥4 out of 5). Apps were then independently reviewed using an author-designed extraction tool.

**Results:**

Overall, 27 apps were included in this review (Google Play Store: 16/27, 59%, and App Store: 11/27, 41%). Two-thirds of the apps provided some information relating to the frequency, intensity, time, and type principles of exercise; only 11% (3/27) provided this information in line with current evidence-based guidelines. Approximately one-third of the apps provided information about contraindications to exercise during pregnancy and referenced the supporting evidence. None of the apps actively engaged in screening for potential contraindications. Only 15% (4/27) of the apps collected information about the user’s current exercise behaviors, 11% (3/27) allowed users to personalize features relating to their exercise preferences, and a little more than one-third provided information about developer credentials.

**Conclusions:**

Few exercise apps designed for pregnancy aligned with current evidence-based physical activity guidelines. None of the apps screened users for contraindications to physical activity and exercise during pregnancy, and most lacked appropriate personalization features to account for an individual’s characteristics. Few involved qualified experts during the development of the app. There is a need to improve the quality of apps that promote exercise in pregnancy to ensure that women are appropriately supported to engage in exercise and the potential risk of injury, complications, and adverse pregnancy outcomes for both mother and child is minimized. This could be done by providing expert guidance that aligns with current recommendations, introducing screening measures and features that enable personalization and tailoring to individual users, or by developing a recognized system for regulating apps.

## Introduction

### Background

Physical activity during pregnancy promotes maternal, fetal, and neonatal health [1]. The health benefits of prenatal physical activity include reduced risk of excessive gestational weight gain, gestational diabetes, pre-eclampsia, delivery complications, preterm birth, newborn complications, and postpartum depression [1]. As such, guidelines for physical activity and exercise during pregnancy recommend that all pregnant women without contraindications (in which the benefits of physical activity and exercise may be outweighed by risks associated with a medical condition) should undertake regular physical activity comprising at least 150 minutes of moderate- to vigorous-intensity aerobic activity each week, along with the incorporation of regular muscle-strengthening exercises (including pelvic floor exercises) [1-3]. Guidelines also identify safety considerations for physical activity and exercise in pregnancy, including absolute and relative contraindications to commencing (previously inactive women) or continuing (previously active women) activity, warning signs and symptoms to stop activity, and exercises to avoid [1,2].

Despite these recommendations and increased interest in health behaviors during pregnancy [4-6], few pregnant women achieve adequate physical activity and exercise [7-10]. A cohort study involving 3482 Norwegian women reported that only 14.6% of the pregnant women followed current guidelines for physical activity during pregnancy at 17-21 weeks’ gestation [7]. One reason for such low adherence rates may be that women are uncertain how to engage safely in physical activity and exercise during this life stage [11-13]. Furthermore, women may receive limited or inaccurate advice on physical activity and exercise participation from health care providers [14-16], prompting them to seek out their own additional information or resources, often from internet communication technologies such as the internet and mobile apps.

The rapid global rise of internet communication technology provides many pregnant women with access to health information, including physical activity and exercise advice, outside of the traditional relationship with a health care provider [17-19]. For instance, in a cross-sectional survey of 293 pregnant US women, a little less than half (44%) had sought information on physical activity through the internet [20]. Similarly, the ubiquity of smartphone ownership in both high- and middle-income countries now allows most pregnant women to use mobile apps as a source of health information [18]. For example, a cross-sectional survey of 410 pregnant Australian women reported that almost three-quarters (73%) used at least one pregnancy app [21]. Although many women seek pregnancy-related information through web-based sources, few discuss this information with their health care providers [18,22].

Interestingly, there are more apps available for pregnancy than for any other medical topic [23]. This is of concern because health-related apps have previously been identified for their potentially inaccurate content and poor quality [22,24,25]. A review of the quality of popular physical activity apps for the general population found that only 18% of the 65 included apps were based on consultation with an expert (eg, medical professional, fitness expert, or behavior change specialist) or on a peer-reviewed study [26].

Furthermore, despite it being well established that behavior change techniques (BCTs) are fundamental to supporting lifestyle change [27], a recent review found that apps designed to promote physical activity and exercise during pregnancy scarcely incorporated BCTs that have demonstrated efficacy for physical activity behavior change (eg, prompt review of behavioral goals) during pregnancy on their platform [27,28]. Another review of the quality and perceived impact of apps designed to address physical activity and exercise during and after pregnancy, which used the Mobile Application Rating Scale, reported that none of the 54 included apps specified goal setting, despite research showing goal setting to be one of the most effective BCTs among pregnant and postpartum women [29].

Although mobile apps are ideally placed to provide easily accessible information for pregnant women to support physical activity and exercise participation, there is also concern over the apps’ safety and lack of regulation of content [18]. Furthermore, it is unclear whether commercial apps on physical activity and exercise during pregnancy align with current evidence-based recommendations [18,22,23].

### Objective

Therefore, the aims of this study are to examine (1) alignment of the content with evidence-based recommendations for physical activity and exercise in pregnancy (ie, screening practices; physical activity and exercise prescription, including exercise frequency, intensity, time, and type (FITT principle); exercise considerations; and warning signs and symptoms to stop activity during pregnancy); (2) delivery, format, and features of physical activity and exercise instruction; and (3) credentials of the app developers.

## Methods

### Methodological Approach

The methodological approach used in this study was informed by previous app reviews [28,30,31] that explored app quality, features, and BCTs among apps designed to (1) improve diet, physical activity, and sedentary behavior in children and adolescents [31]; (2) provide nutritional advice to pregnant women [30]; and (3) promote prenatal physical activity and exercise [28].

### Search Strategy

Systematic searches were conducted in the Australian App Store and Google Play Store in October 2020. Apps were identified using combinations of search terms relevant to pregnancy and exercise or physical activity (see Multimedia Appendix 1 for detailed search term combinations and strategy). Each search term combination was entered individually in the App Store and Google Play Store databases without any specified search categories, and search results were automatically ordered by the respective app store’s *relevance* algorithm. That is, ordered by text relevance (ie, search term relevance to app title, keywords, and primary category) and user behaviors (ie, number of downloads and user ratings).

### Inclusion Criteria and Selection Process

The apps underwent an initial screening and were included if the title and brief description of the app suggested a focus on physical activity or exercise during pregnancy, was available in English, not used as a studio-booking tool, and did not require any external devices (eg, Kegel device, activity monitor, or physical books). App characteristics, including app name, developer, version, store (App Store or Google Play Store), category, year of last update, cost, and average user rating were then extracted from the remaining apps (Table 1). The apps then underwent a secondary screening for inclusion by 2 independent reviewers (KA and SC), as per best practice for systematic reviews [32], and were deemed eligible for inclusion in this review if (1) they had been published or updated since 2018 (to ensure currency), (2) they were free to download and did not require an immediate paid subscription, and (3) they had an average user rating of ≥4 out of 5 because apps with higher standardized user ratings are more frequently downloaded [33]. Any disagreements in the screening process were resolved by consensus.

Each of the eligible apps was then independently reviewed by 2 of the 22 reviewers (ie, the authors, who are recognized as having expertise in physical activity and exercise and pregnancy as well as app reviews, including researchers, health professionals, and clinicians such as exercise physiologists). This review involved downloading the app, user testing, and assessing app features and quality criteria. If an app offered a free trial of a premium version, the reviewers were asked to assess the content delivered in the free trial. If no free trial was offered or if the app did not have a premium version, the standard (free) content was assessed. Freemium content (ie, extra content at a cost) was not assessed, and apps requiring immediate paid subscription (ie, no free trial) were excluded. The reviewers were provided with fictitious profiles to be used when personal information was required as well as instructions to gain familiarity with the app before data extraction (Multimedia Appendix 2). In cases of disagreement between the 2 reviewers, a third reviewer was assigned to provide an additional review, specifically focusing on the item of disagreement, to arrive at a majority decision.

**Table 1:** Characteristics of the apps included in this review (N=27).

Store and app ID		App name	Developer	Costing^a^	Version	Update	Rating, mean	Ratings, n
**App Store**								
	1	Baby2Body: Pregnancy Wellness	Baby2Body Limited	Subscription	3.5.7	2020	4.6	534
	2	Emily Skye FIT: Workout App	Loup Pty Ltd	Subscription	1.18.0	2020	4.1	63
	3	Juna: Pregnancy Workouts	Juna Media LLC	Freemium	1.9.5	2020	4.2	5
	4	Moms Into Fitness	Moms Into Fitness, Inc	Subscription	5.801.1	2020	5	1
	5	Pregnancy +	Health & Parenting Ltd	Freemium	5.11	2020	4.8	7400
	6	Prenatal Yoga | Down Dog	Yoga Buddhi Co	Subscription	5.2.2	2020	4.9	1100
	7	Tips for Pregnant: Hello Belly	HelloBaby, Inc	Freemium	2.0.9	2020	4.1	212
	8	Tone It Up: Workout & Fitness	Tone It Up, LLC	Subscription	2.4.6	2020	4.7	127
	9	U Pilates: Workouts & Exercise	U Pilates Ltd	Freemium	3.19.2	2020	5	1
	10	Yoggy: pregnancy yoga workouts	Millefeuille Agency	Freemium	2.7	2020	4.6	29
	11	YogiBirth: Pregnancy Yoga App	YogiBirth Pty Ltd	Freemium	1.1.3	2020	4.9	196
**Google Play Store**								
	12	9MonthsGuide	9MonthsGuide Team	Free	4	2019	4.4	511
	13	Happy Pregnancy App	Dr Sachin Gothi (ObGyn)	Free	7	2019	4.7	211
	14	Healthy pregnancy tips	My Apps Studio	Free	1	2019	5	180
	15	I’m Pregnant: Pregnancy Week By Week	BabyJoyApp	Freemium	4	2018	4.7	36,000
	16	Jillian Michaels: The Fitness App	EM Digital LLC	Subscription	3.9.9	2020	4.5	4000
	17	Kegel Exercises for Men & Women: A How-to Guide	MasterpieceApps	Free	3.1	2020	4.2	22
	18	Move Your Bump	Move Your Bump	Freemium	5.900.1	2020	4.7	14
	19	My pregnancy calendar app: baby countdown timer	BabyInside	Freemium	2.0.9	2020	4.6	1000
	20	My Pregnancy Journey	My Pregnancy Journey	Freemium	1.0.9	2019	4.3	20
	21	pregnancy calendar	ruthie apps	Free	4.11.7.0	2018	4.2	37
	22	Pregnancy Companion: Week by Week Tracking	Healthcare Apps	Free	1.8	2020	4.3	253
	23	Pregnancy Exercise and Workout at Home	Pregnur Apps	Free	2.0.9	2020	4.6	3000
	24	Pregnancy Exercises	B6Squad Dev.	Free	6	2020	4.1	123
	25	Pregnancy Guide	ARVIRA DEV	Freemium	1.12	2019	4.6	7000
	26	Pregnancy Guide App	EllStudiosApp	Free	3	2020	4.4	164
	27	Pregnant. Pregnancy by week. Pregnancy calendar	rusakov77	Free	3.84	2019	4.4	190

^a^Subscription: free presubscription trial; freemium: offers premium content through in-app purchases; free: all content freely accessible.

### Data Extraction

A tool was specifically created for data extraction purposes based on 2 of the most recently released evidence-based recommendations for physical activity and exercise during pregnancy [1,2]. Before the full data extraction process, 6 reviewers piloted the data extraction tool with 5 apps. Interrater consistency and feedback were considered during refinement of the extraction tool (see Multimedia Appendix 2 for final extraction tool detail).

All 22 reviewers used the final extraction tool to assess whether the app asked the user for any personal information about themselves (eg, age, height, and weight) or about their current pregnancy (eg, due date, current trimester, and singleton or multiple pregnancy). They also assessed the alignment of app content with evidenced-based recommendations, including disclaimers and terms and conditions; contraindication screening and information; exercise behavior, intention, and preferences; physical activity and exercise content (FITT principle of exercise); contraindications to physical activity and exercise during pregnancy; safe pregnancy exercises or warning signs and symptoms to stop physical activity and exercise [1,2]; how the information was delivered (through still image, video, audio, text, etc); opportunities for users to modify exercises; and whether the app provided validation or references for its content. In addition to dichotomous responses (yes or no) and multiple-choice selections, the reviewers were provided with open-response textboxes to elaborate on their review or add further information. Any disagreements among the reviewers were resolved in consultation with a third reviewer.

### Statistical Analyses

Descriptive statistics on the collated responses were derived (mean, SD, and frequency) using RStudio. Open-ended responses were summarized into *Other* categories for each representative item of interest.

## Results

### App Selection

**Figure 1:**
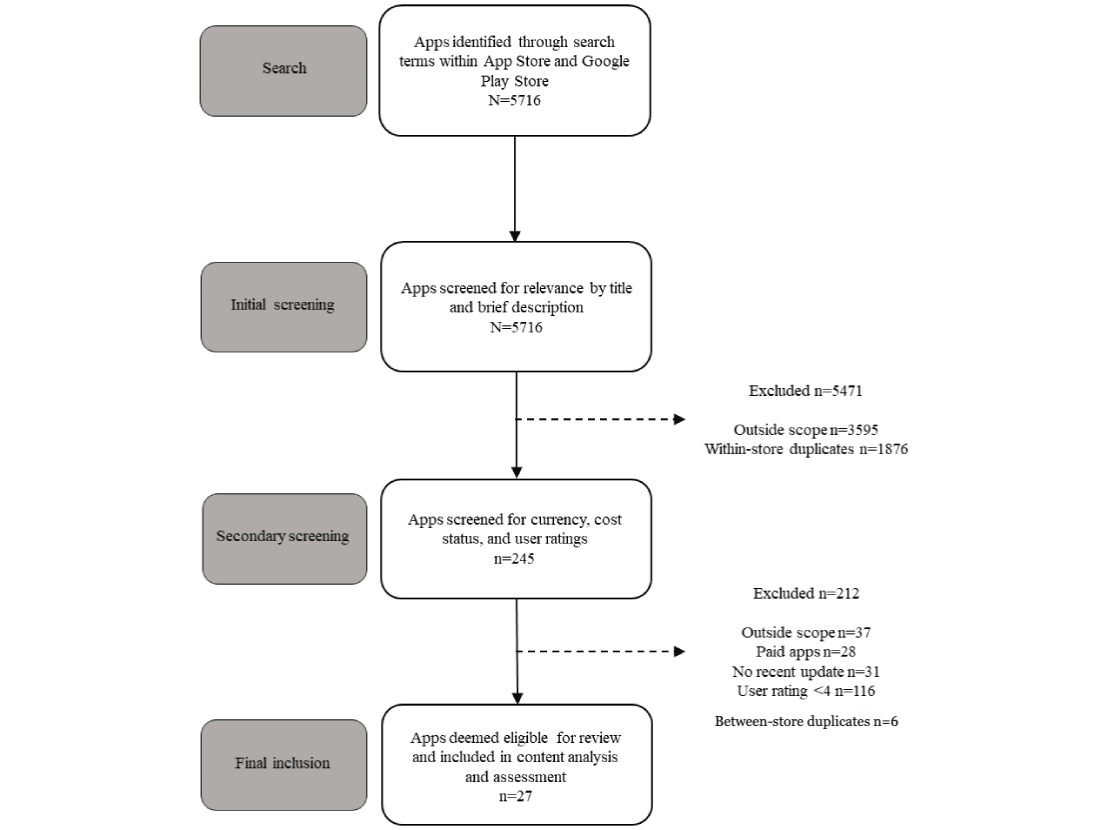
Flowchart of the app selection process.

A flowchart of the app selection process is presented in Figure 1. App Store and Google Play Store searches resulted in a total of 5716 apps for screening. The initial screening involved excluding 95.71% (5471/5716) of the apps that did not focus on physical activity or exercise in their title and description, were not available in English, required a studio-booking system, or required external devices. Of the remaining 245 apps, a second screening further excluded 212 (86.5%) apps based on the inclusion criteria and 6 (2.5%) apps that were between-store duplicates, leaving a total of 27 (11%) apps targeting exercise during pregnancy for inclusion in the final sample for data extraction and content analysis.

### App Characteristics

Of the 27 reviewed apps, 16 (59%) were accessed through Google Play Store and 11 (41%) through the App Store (Table 1). All apps were free to download; of the 27 apps, 10 (37%) offered all content for free, 6 (22%) offered a free trial of the premium subscription, and 11 (41%) offered restricted content in the absence of a paid premium version or paid subscription. The average star rating in the app stores at the time of downloading the apps was 4.54 of 5 (SD 0.29; range 4.1-5; skew 0.009; median 4.6, IQR 0.5), with a wide range of the number of users rating each app (range 1-36,000 user ratings; mean 2310.85, SD 7026.49; skew 4.09; median 190, IQR 734). All the included apps primarily prescribed structured exercise (ie, intentional and predetermined activity sessions), rather than lifestyle physical activity (ie, recreational activity incorporated into daily living, eg, gardening). Almost half of the apps were considered general pregnancy apps that provided a range of pregnancy information, including exercise-specific content; 33% (9/27) were considered pregnancy-specific exercise apps; and 19% (5/27) were considered general exercise apps that offered a section specifically for pregnancy exercise content. Of the 27 apps, 18 (67%) presented users with several types of exercises (eg, a variety of aerobic, strength, pelvic floor, and flexibility exercises), whereas the remaining 9 (33%) offered users only 1 specific type of exercise (eg, only yoga or only weightlifting). Characteristics of the 27 apps included in this review are presented in Table 1.

### Alignment of Content With Guidelines for Physical Activity and Exercise in Pregnancy

#### Disclaimers and Terms and Conditions

Of the 27 apps, 19 (70%) presented users with a disclaimer or terms and conditions absolving the app developers of liability (ie, user participation is at their own risk and the app is not responsible for any adverse outcomes that may occur when using the app or as a result of using the app). Users were required to actively agree to the terms and conditions in 37% (10/27) of the apps, whereas 19% (5/27) required users to agree to a disclaimer and 11% (3/27) asked users to actively agree to both terms and conditions as well as a disclaimer. Within the apps’ disclaimer or terms and conditions, 67% (18/27) of the apps recommended that women seek medical clearance before commencing exercise during pregnancy (Multimedia Appendix 3). However, only 4% (1/27) specifically asked users to confirm whether they had obtained approval or clearance from their professional health care provider to engage in exercise while pregnant.

#### Screening for Contraindications to Exercise During Pregnancy

None of the apps specifically asked the user whether they had any absolute (eg, ruptured membranes, pre-eclampsia, or preterm labor) or relative (eg, symptomatic anemia or history of spontaneous miscarriage) contraindications to exercise during pregnancy.

Information about contraindications to exercise during pregnancy was limited. Of the 27 apps, 17 (63%) made no reference to contraindications, whereas the remaining 10 (37%) noted ≥1 recognized contraindications. The most frequently noted contraindications were history of spontaneous miscarriage, premature labor, or fetal growth restrictions (8/27, 30%), placenta previa (7/27, 26%), and persistent second- or third-trimester bleeding (7/27, 26%; Table 2 and Multimedia Appendix 4). Contraindications and related information were accessible not only within app information tabs and sections or within the exercise instructions and demonstrations, but also in some disclaimers and terms and conditions. Other medical issues of concern, such as chronic toxicities and infectious diseases, were noted in 15% (4/27) of the apps.

**Table 2:** Information about contraindications provided in the apps (N=27).

Contraindication	Apps providing information, n (%)
History of spontaneous miscarriage, premature labor, or fetal growth restriction	8 (30)
Placenta previa	7 (26)
Persistent second- or third-trimester bleeding	6 (22)
Serious cardiovascular, respiratory, or systemic disorder	5 (19)
Incompetent cervix	5 (19)
Ruptured membranes or premature labor	5 (19)
Pre-eclampsia	5 (19)
Mild to moderate cardiovascular or chronic respiratory disease	4 (15)
Symptomatic anemia	3 (11)
Evidence of intrauterine growth restriction	2 (7)
Multiple gestation (eg, triplets or higher number)	2 (7)
Pregnancy-induced hypertension	2 (7)
Poorly controlled type 1 diabetes, hypertension, or thyroid disease	1 (4)
Twin pregnancy after the 28th week	1 (4)

#### Exercise Behaviors, Intentions, and Preferences

Of the 27 reviewed apps, 4 (15%) asked the user questions about their current exercise behaviors and 4 (15%) asked the user questions about their current intentions for exercise (Multimedia Appendix 4). When closing the app and reopening it or logging back into the account, only 7% (2/27) of the apps asked the user whether they wanted to provide or update any personal information or exercise preferences (eg, weight, height, number of sessions per week, type of exercise session, and available equipment).

#### FITT Principle of Exercise

Of the 27 apps, only 5 (19%) recommended accumulating at least 150 minutes of exercise per week, whereas 6 (22%) specified the amount of exercise that should be accumulated per day. Of the 27 apps, 13 (48%) provided information on exercise frequency and 14 (52%) recommended exercise intensity, whereas only 4 (15%) provided information about the duration of each exercise session. All apps recommended at least one type of exercise (Table 3 and Multimedia Appendix 5).

Of the 27 apps, 13 (48%) recommended exercise frequency; all of them noted that exercise should be performed on most, if not all, days of the week in accordance with current evidence-based guidelines for physical activity and exercise during pregnancy. However, of these 13 apps, only 3 (23%) also suggested at least two sessions of resistance-based exercise per week.

**Table 3:** Recommended frequency, intensity, time, and types of exercises during pregnancy in the reviewed apps (N=27).

				Values, n (%)
**Frequency of exercise**				
		Exercise on most, if not all, days of the week	13 (48)	
		Two sessions of resistance-based exercise per week	3 (11)	
**Intensity of exercise**				
		Light intensity	10 (37)	
		Moderate intensity	10 (37)	
		Vigorous intensity	4 (15)	
**Intensity measurement tool**				
		Talk Test to judge intensity of exercise	5 (19)	
		Borg Rating of Perceived Exertion Scale	4 (15)	
		Heart rate zones (based on age and fitness level)	3 (11)	
**Total time**				
		Accumulate at least 150 minutes of exercise per week	5 (19)	
**Exercise duration bouts (minutes)**				
		Exercise for 30 minutes per day	6 (22)	
		Exercise for at least 15 minutes per session	4 (15)	
		Break up exercise into small bouts	3 (11)	
**Type of exercise**				
	**Aerobic**			
		Walking or jogging or running	11 (41)	
		Swimming	5 (19)	
		Cycling	3 (11)	
	**Muscle strengthening**			
		Pelvic floor or Kegel exercises	19 (70)	
		Strength training (resistance or weight)	14 (52)	
	**Other**			
		Yoga	21 (78)	
		Stretching or flexibility	19 (70)	
		Pilates	11 (41)	
		High-intensity interval training	5 (19)	

Of the 27 apps, 14 (52%) recommended exercise intensity. Of these 14 apps, 10 (71%) recommended that users engage in light-intensity physical activity, whereas 10 (71%) recommended moderate-intensity physical activity (6/14, 43% suggested both light and moderate intensities). Specifically, of the 14 apps, only 4 (29%) recommended moderate- to vigorous-intensity physical activity in accordance with current guidelines [1,2]. Furthermore, of the 14 apps, only 6 (43%) suggested a measurement tool to monitor exercise intensity. These included the Talk Test (5/14, 36%) [34], the Borg Rating of Perceived Exertion Scale (4/14, 29%) [35], and heart rate zones (3/14, 21%) [36].

Of the 27 apps, only 9 (33%) recommended a total weekly duration of exercise, although guidelines for some physical activity and exercise during pregnancy suggest that women should work toward accumulating a total of 150-300 minutes of physical activity and exercise per week [1,2]. Of the 9 apps that did recommend a total duration, 5 (56%) recommended at least 150 minutes per week, whereas 6 (67%) recommended at least 30 minutes per day. Of these 9 apps, 3 (33%) also advised women that they could break up their exercise into smaller *bouts,* if required, in accordance with current evidence-based guidelines for prenatal physical activity and exercise for pregnant women, which recommend that women progressively build their activity levels toward meeting the guidelines [1,2]. Conversely, of the 9 apps, 4 (44%) specifically suggested that exercise should be accumulated in bouts of at least 15 minutes each, which does not align with current guidelines for physical activity and exercise for pregnant women [1,2].

All apps recommended at least one type of exercise, with yoga (21/27, 78%), stretching or flexibility exercises (19/27, 70%), and pelvic floor or Kegel exercises (19/27, 70%) being the most frequently recommended. Aerobic exercises such as walking, jogging, and running (11/27, 41%); swimming (5/27, 19%); and cycling (3/27, 11%) were less frequently recommended. Muscle-strengthening exercises were recommended in 52% (14/27) of the apps, whereas high-intensity interval training (HIIT) was recommended in 19% (5/27) of the apps.

Of the 27 apps, only 3 (11%) provided accurate advice in full accordance with current evidence-based guidelines for physical activity and exercise during pregnancy, in relation to the FITT principle of exercise. Progression of exercise (Multimedia Appendix 5) was included in 37% (10/27) of the reviewed apps, with the most recommended progressions focused on steady progression toward physical activity and exercise guidelines (9/27, 33%) and modifications to exercise as the pregnancy progresses (8/27, 27%).

#### Exercises Considered Safe, Exercises to Avoid, and Other Exercise Considerations

Of the 27 apps, 19 (70%) listed exercises that are considered safe during pregnancy (Table 4 and Multimedia Appendix 6), such as pelvic floor exercises (15/19, 79%), aerobic exercises (13/19, 68%), and muscle-strengthening exercises (13/19, 68%). Pregnancy-specific classes were less frequently listed (8/19, 42%).

**Table 4:** Safe exercises, exercises to avoid, and warning signs or symptoms to stop exercise during pregnancy as provided in the apps (N=27).

		Values, n (%)
**Exercises considered safe**		
	Pelvic floor exercises	15 (56)
	Aerobic physical activity and exercise (walking, cycling, and swimming)	13 (48)
	Muscle-strengthening exercises using body weight, weights, or resistance bands	12 (44)
	Pregnancy-specific classes	8 (30)
**Exercises considered unsafe**		
	Risk of falling (eg, exercise requiring balance, coordination, and agility)	12 (44)
	Risk of contact or collision (eg, basketball and soccer)	9 (33)
	Long periods of laying in the supine position	9 (33)
	Heavy lifting (weights or lifting weight overhead)	6 (22)
	Significant changes in pressure (eg, skydiving and scuba diving)	5 (19)
	Exercise at high altitude	2 (7)
	Long periods of standing still	1 (4)
**Warning signs or symptoms to stop exercise**		
	Persistent dizziness or feeling faint, which does not resolve with rest	12 (44)
	Persistent excessive shortness of breath, which does not resolve with rest	11 (41)
	Regular painful uterine contractions	9 (33)
	Vaginal bleeding	8 (30)
	Heat stress or hyperthermia in first trimester	8 (30)
	Chest pain	7 (26)
	Persistent loss of fluid from the vagina (possible ruptured membrane)	7 (26)
	Inadequate nutrition	7 (26)
	Dehydration	6 (22)
	Severe headache	5 (19)

Of the 27 apps, only 13 (48%) listed specific exercises to avoid or physical activities that are recognized as unsafe during pregnancy. These included physical activities that increase the risk of falling (12/13, 92%), physical activities with an increased risk of contact or collision (9/13, 69%), and exercise in the supine position (9/13, 69%). Other exercise activities such as strong stretches, twists and backbends, skiing, skating, and bungee jumping were less frequently mentioned as physical activities to avoid.

Further evidence-based considerations relating to exercise during pregnancy were apparent in 67% (18/27) of the apps. The most common of these was the importance of staying well hydrated (15/27, 56%), whereas 33% (9/27) of the apps recommended that women wear appropriate clothing during exercise (Multimedia Appendix 6).

#### Warning Signs and Symptoms to Stop Activity During Pregnancy

Of the 27 apps, 18 (67%) listed warning signs and symptoms for stopping exercise during pregnancy in accordance with current evidence-based guidelines for physical activity and exercise during pregnancy (Table 4 and Multimedia Appendix 6). Persistent dizziness or feeling faint (12/18, 67%) and persistent excessive shortness of breath that does not resolve with rest (11/18, 61%) were the most common signs and symptoms listed in the reviewed apps.

### Delivery, Format, and Features of Exercise Instruction

Of the 27 apps, 18 (67%) presented exercise as a series of individual exercises and 12 (44%) presented exercise as a series of workouts (with predetermined sequences of exercises; Multimedia Appendix 7). Of the 27 apps, 4 (15%) provided a combination of individual exercises and workouts, whereas 3 (11%) provided a combination of workouts and programs (predetermined sequences of workouts); only 1 (4%) app provided a 3-way combination of individual exercises, workouts, and programs. The most common format used by the apps to deliver exercise instruction was written cues (22/27, 81%), followed by video demonstrations (16/27, 59%), spoken cues (14/27, 52%), and finally, illustrations or still pictures to demonstrate the exercise (12/27, 44%). Of the 27 apps, 22 (81%) provided a combination of ≥2 of these instructional formats, 12 (44%) provided a combination of ≥3 instructional formats, and 3 (11%) provided a combination of all 4 instructional formats.

Of the 27 apps, 18 (67%) provided up-front details of the exercise session to help users decide which exercise to perform (Multimedia Appendix 7). These details most commonly included the duration of the exercise session (17/18, 94%), the type of exercise session (13/18, 72%), the suggested trimester in which to perform the exercise (11/18, 61%), and the equipment required to perform the exercise (10/18, 56%). Of the 18 apps, only 1 (6%) provided information up-front regarding the FITT principle of exercise, experience level, equipment, and trimester to fully inform the user of the exercise details. Moreover, only 17% (3/18) of these apps allowed users to modify the FITT parameters; however, none of these apps provided advice or feedback about exercise modifications.

### Expertise and Credentials of App Developers

Of the 27 apps, only 6 (22%) specified the app developers’ formal qualifications and 8 (30%) provided information to imply the developers’ expertise or credibility (4/27, 15%, apps provided information on both formal and experiential credibility; Table 5 and Multimedia Appendix 7). Developer qualifications included master’s in kinesiology, master’s in exercise science, obstetrician, medical degree, master’s in sports medicine, sports psychologist, qualified fitness instructor, personal trainer, master’s in biology, Pilates instructor, midwife, and childbirth educator. The expertise or credibility of the app developer was often implied by the developer or instructor stating that they had years of practical experience in prenatal and postnatal support domains or had been through pregnancy themselves. Apps reporting formal qualifications or experiential credibility within their development team were more likely to support their content with recognized references. However, there was no difference in alignment of the content with evidence-based recommendations for physical activity and exercise in pregnancy.

**Table 5:** Developers’ recognized credentials, experiential credibility, and referenced sources of information as provided in the apps (N=27).

		Apps providing developer information, n (%)
**Developers’ credentials and credibility**		
	Implies developers’ credibility or experience	8 (30)
	Specifies developers’ recognized qualifications	6 (22)
**Reference to recognized sources of information**		
	Government guidelines (exercise guidelines that incorporate pregnancy and guidelines for exercise during pregnancy)	7 (26)
	Academic literature	6 (22)
	Obstetrics-related guidelines	5 (19)

### Sources Used to Guide App Content

Of the 27 apps, only 9 (33%) referenced a recognized or high-quality source of information (Table 5 and Multimedia Appendix 7), such as government (7/27, 26%) or obstetrics-oriented guidelines (5/27, 19%). In addition, 22% (6/27) of the apps referred to academic literature. However, of the 27 apps, only 1 (4%) provided a reference list to support the content provided.

## Discussion

### Principal Findings

This is the first review of apps that promote exercise in pregnancy to examine (1) alignment of the content with evidence-based recommendations for physical activity and exercise in pregnancy; (2) delivery, format, and features of exercise instruction; and (3) credentials of the developers. Specifically, we identified a lack of alignment with current evidence-based recommendations for physical activity and exercise [1,2], particularly relating to screening of contraindications to exercise during pregnancy and to exercise prescription (FITT principle). In addition, few apps provided appropriate user opportunity to tailor the apps to their individual exercise needs, listed the sources of information used to guide content, or showed the credentials of the app developers. The results of this review highlight a need for improved regulation of the content of apps that promote exercise during pregnancy.

Current evidence-based guidelines for physical activity and exercise during pregnancy clearly indicate absolute and relative contraindications for participation in physical activity and exercise [1,2]. Women with absolute contraindications to physical activity and exercise during pregnancy are advised to avoid moderate to vigorous activity because the benefits of physical activity and exercise are outweighed by the risks [1]. Furthermore, those with relative contraindications are advised to discuss the advantages and disadvantages of physical activity and exercise, as well as potential modifications, with an appropriately qualified health care provider (such as their obstetric care provider) before participation [1]. Yet, almost two-thirds of the apps made no reference to absolute or relative contraindications and none included any kind of screening for pregnant women to identify contraindications. This is of concern, given that many pregnant women are increasingly turning to apps for guidance and support rather than relying on face-to-face information that they would traditionally receive from their health care providers [18]. Specifically, no users were asked to enter information or *check* contraindications from a provided list at any stage while engaging with the app. Although two-thirds of the apps advised users to seek medical clearance before commencing physical activity, none acknowledged (or considered) that contraindications to exercise can occur at any time throughout pregnancy. Thus, active screening for contraindications (using a simplified screening tool or method to limit impact when accessing the app) should be a feature of all apps and should be repeated with each user interaction frequently throughout the pregnancy. Warning signs and symptoms to stop physical activity and exercise are also clearly listed in current evidence-based guidelines for physical activity and exercise during pregnancy [1,2]. Despite this, only half of the apps provided educational information on these signs and symptoms. As such, women may continue to engage in physical activity and exercise while also risking the health and well-being of their pregnancy because they are unaware of the signs and symptoms to cease activity. The inclusion of a simple checklist listing the signs and symptoms to stop activity may help to prevent potential adverse events, while further providing evidence-based information to users. Furthermore, although 67% (18/27) of the apps included *Terms and Conditions* or a *Disclaimer* to encourage women to seek clearance from a health care provider before commencing exercise, only 44% (12/27) required acceptance of, or agreement with, the conditions or disclaimer. In fact, the American College of Obstetricians and Gynecologists recommends that a thorough clinical examination be conducted before commencing an exercise program to ensure the safety and well-being of the pregnancy and to check that pregnant women do not have any medical reasons to avoid exercise during this unique life stage [37].

Although most of the apps provided some level of information on frequency, intensity, total time (as well as duration of session bouts), or type of exercise, only 11% (3/27) of the apps did so in line with current guidelines. Instead, most of the reviewed apps recommended light-intensity exercise, although guidelines recommend that women engage in moderate- to vigorous-intensity physical activity and exercise [1,2] to achieve the greatest health benefits. In addition, 19% (5/27) of the apps recommended HIIT, which typically consists of alternating periods of vigorous- to high-intensity aerobic exercise with light recovery exercise or no exercise [38]. Although preliminary research suggests that this type of training seems to be well tolerated among a small cohort (N=14) of active pregnant women who engaged in a single session of HIIT [39], there is insufficient evidence to suggest that HIIT is safe during pregnancy. As such, current evidence-based guidelines do not recommend it [1,2]. Moreover, only 22% (6/27) of the apps provided women with advice on how to measure and monitor exercise intensity.

Very few apps provided users with up-front information pertaining to the exercise or workout (such as equipment required, duration and intensity of workout, and experience level), therefore limiting the users’ ability to make an informed decision regarding the appropriateness of the exercise and or workout. By providing this important information up-front, the user can make an informed decision about the appropriateness of the exercise or workout, while taking into account their own exercise behaviors, experiences, and current pregnancy status.

Given that only one-third of the reviewed apps referred to relevant and recognized expert sources of information (ie, physical activity and exercise guidelines or peer-reviewed literature) within the app, it is not surprising that few aligned their exercise prescription with current evidence-based guidelines. These findings are similar to other app reviews that have evaluated the accuracy of app content [18,40]. For instance, Subhi et al [40] conducted a review of 52 studies (N=6520 apps) to examine expert involvement and adherence of app content to medical evidence in medical mobile phone apps. They found 30 studies (which included 3051 apps) that explored adherence to medical evidence in app content. In 17 of these studies, none of the app content was found to accurately reflect, or adhere to, medical evidence. The remaining 13 studies found that 10%-87% of the apps’ content accurately reflected medical evidence. Moreover, only 5 of these 13 studies reported complete adherence and alignment of app content to medical evidence in more than 50% of the assessed apps [40].

Few apps collected users’ individual activity characteristics, thus limiting the ability to appropriately tailor exercise prescriptions. Only half the included apps collected information about the current pregnancy, and only 15% (4/27) of the apps asked about current exercise behaviors. Given that these characteristics are fundamental to safe and appropriate exercise prescription in pregnancy [41], this may lead to women engaging in inappropriate physical activity. Users’ individual characteristics, including their current physical activity and exercise behaviors and medical history, are essential if apps are to support appropriate and individualized physical activity and exercise prescriptions that align with evidence-based guidelines [1,2].

Finally, only 22% (6/27) of the apps provided the formal qualifications of the app developers. This is consistent with previous reviews of the involvement of experts in app development [18,26]. For example, a recent review of popular physical activity apps for the general population found that only 12 of the 65 reviewed apps reported expert (ie, fitness expert, behavior change specialist, and medical professional) involvement in the development of the app [26]. Similarly, in the review by Subhi et al [40] of 52 studies, 28 studies assessed 3852 medical apps for expert involvement. The review found that 9%-67% of the apps reported expert involvement to some extent [40]. In a review of 129 urology apps to identify predictors of the number of urology app downloads, the explicit participation of urologists in app development was found to be likely to enhance the apps’ chances to have a higher number of downloads [42], signifying the potential to improve app quality without compromising app popularity. However, it is important that those engaged in the app development process are up to date with current evidence-based guidelines because this review suggests that, despite involving those with formal qualifications or experiential credibility in the development process, the content was no more aligned with evidence-based recommendations for physical activity and exercise during pregnancy than in apps that did not report expert involvement.

### Implications of Findings and Future Directions

It is clear that the rapid proliferation of apps targeting exercise in pregnancy has not been accompanied by a focus on ensuring user safety, adherence with evidence-based guidelines, and appropriateness of content. Given that health apps remain largely unregulated [18], there is a need for knowledge translation and implementation science to improve future practice. This should involve collaboration with stakeholders (ie, pregnant women) to ensure user satisfaction and with app developers, health care providers, and researchers to ensure that apps reflect evidence-based guidelines [43,44]. A key area of focus should be the incorporation of thorough pre-exercise screening practices that enable appropriate tailoring of exercise prescription to each user’s unique individual characteristics. This may mean better incorporation of screening through the individual apps themselves or the creation of a stand-alone pre-exercise screening app to integrate with individual platforms. Apps that demonstrate collaboration or review by experts in physical activity and exercise during pregnancy could be recognized or registered through an app directory [43,44]. However, app directories to date do not include physical activity and exercise apps for pregnant women. Instead, they tend to focus on medical services such as streamlining communication among patients, providers, and their caregivers, allowing 24/7 management of a patient’s condition and prescriptions, and improving organizational workflow. Moreover, none of the apps included in this review were developed by 1 of the 9 companies selected by the US Food and Drug Administration to participate in the development of the Software Pre-Cert Pilot Program [45]. Such regulation or certification of apps would help to ensure user safety [43,44]. However, it remains unclear whether commercial apps designed to target physical activity behaviors among pregnant women will require approval through this precertification program because they may not be recognized as “software intended to be used for one or more medical purposes that perform these purposes without being part of a hardware medical device” [45]. Thus, many of the apps, such as those included in this review, will likely continue to be developed in an unregulated commercial market.

### Strengths and Limitations

The strengths of this review are that it included a systematic search for apps on both the App Store (Apple) and Google Play Store and that all reviewers were experts in physical activity and exercise in pregnancy. As per best practice for conducting systematic reviews [32], 2 independent reviewers (KA and SC) extracted data from each app using a pre-established and piloted extraction tool. Although app reviews are widely accepted for providing a snapshot in time, this approach may be considered a limitation, albeit unavoidable because apps require updating every 2 years. A decision was made to only include apps with freely available content in this review and those with a user rating of ≥4 out of 5. This was based on previous research that shows that few consumers are willing to pay for health apps and that consumers are more likely to download apps with a higher user rating [46]. However, the exclusion of numerous apps from this review may be considered a limitation because the findings cannot be generalized to apps that require immediate paid subscription or apps that provide freemium content or apps with user ratings of <4 out of 5. To improve inclusiveness, future studies might consider app exposure rates, download rates, and user comments as part of their inclusion criteria. In addition, if an app was available in both the App Store and Google Play Store, the App Store app was selected for inclusion in the study because fewer reviewers had access to Android devices. Although this is not necessarily a limitation, it should be acknowledged that some apps included in this study may also be accessible on the Google Play Store with different app characteristics.

### Conclusions

Apps are a popular source of information and guidance for health behaviors during pregnancy, including physical activity and exercise. Our results demonstrate that neither do apps provide appropriate screening features to identify potential contraindications to exercise during pregnancy, nor do they provide content in accordance with current evidence-based physical activity guidelines or personalization. Overall, very few apps were found to have been developed by, or in conjunction with, experts (ie, health or medical professionals with expertise in prenatal exercise). This review emphasizes a critical need for development of evidence-based, tailored apps with greater regulation to minimize the potential risk of injury, complications, and adverse pregnancy outcomes for both mother and child.

## Appendix

Multimedia Appendix 1 Search strategy.

Multimedia Appendix 2 Data extraction tool.

Multimedia Appendix 3 Personal information, terms and conditions, and disclaimer.

Multimedia Appendix 4 Contraindications and behavior and intention screening.

Multimedia Appendix 5 Frequency, intensity, time (total time accumulated throughout the week), and type principle of exercise and progression.

Multimedia Appendix 6 Safety, benefits, and considerations.

Multimedia Appendix 7 Delivery, format, and features of exercise instruction.

## Abbreviations

BCT: behavior change technique
FITT: frequency, intensity, time, and type
HIIT: high-intensity interval training
