# Supplementary material for: Evaluating Evidence-Based Content, Features of Exercise Instruction, and Expert Involvement in Physical Activity Apps for Pregnant Women: Systematic Search and Content Analysis
Source: JMIR Mhealth Uhealth. 2022 Jan 19;10(1):e31607. doi: 10.2196/31607 (PMC8811692; doi:10.2196/31607)
Supplement: Multimedia Appendix 1 [file mhealth_v10i1e31607_app1.docx]

**Multimedia Appendix 1: Search Strategy and Screening Results**

*Detailed search terms and criteria used in search and screen phases*

| Search / Screen | Task | Details |
| --- | --- | --- |
| App store search | Timeframe | October 2020 |
|  | Search terms | Pregnancy exercises, pregnancy exercise, pregnancy fitness, pregnancy workout, pregnancy physical activity, pregnant exercises, pregnant exercise, pregnant fitness, pregnant workout, pregnant physical activity, prenatal exercises, prenatal exercise, prenatal fitness, prenatal workout, prenatal physical activity |
|  | Results display | In order of automatic respective app stores’ ‘relevance’ algorithm |
| Initial screening | Timeframe | October – November 2020 |
|  | Inclusion criteria | Suggests a focus on exercise and/or physical activity during pregnancy; Is available in the English language; Is not used as a studio booking tool; Does not require external/additional devices (e.g., Kegel device, activity monitor, hardcopy book, etc.); Removal of within-store duplicate apps |
| Secondary screening | Timeframe | November 2020 |
|  | Inclusion criteria | App description suggests primary focus on exercise and/or physical activity during pregnancy; Is free to download and do not require any immediate payment for subscription (e.g., had no subscription or free pre-subscription trial); Has been updated by developer within previous two years (since 2018); Has average user rating of at least 4 out of 5; Removal of between-store duplicate apps |

*Detailed app search and screen results*

| Search Term | Search Results (n) | | Initial Screening (n) | | Secondary Screening (n) | | |
| --- | --- | --- | --- | --- | --- | --- | --- |
|  | App Store | Google Play | App Store | Google Play | App Store | | Google Play |
| Pregnancy exercise | 143 | 250 | 38 | 112 | 11 | | 22 |
| Pregnancy exercises | 210 | 250 | 32 | 109 |  |  |  |
| Pregnancy fitness | 209 | 250 | 42 | 124 |  |  |  |
| Pregnancy workout | 211 | 250 | 46 | 115 |  |  |  |
| Pregnancy physical activity | 0 | 250 | 0 | 112 |  |  |  |
| Pregnant exercise | 197 | 250 | 38 | 120 |  |  |  |
| Pregnant exercises | 26 | 250 | 17 | 114 |  |  |  |
| Pregnant fitness | 215 | 250 | 39 | 118 |  |  |  |
| Pregnant workout | 190 | 250 | 46 | 108 |  |  |  |
| Pregnant physical activity | 0 | 250 | 0 | 104 |  |  |  |
| Prenatal exercise | 62 | 250 | 37 | 114 |  |  |  |
| Prenatal exercises | 175 | 250 | 44 | 104 |  |  |  |
| Prenatal fitness | 164 | 250 | 50 | 96 |  |  |  |
| Prenatal workout | 164 | 250 | 48 | 97 |  |  |  |
| Prenatal physical activity | 0 | 250 | 0 | 97 |  |  |  |
| Excluded | - | - | 1489 | 2106 | 77 | 135 | |
| Duplicates | - | - | 389 | 1487 | -0* | -6* | |
| Within-store totals | 1966 | 3750 | 88 | 157 | 11 | 16 | |
| Combined Totals | 5716 | | 245 | | 27 | | |

*Between-store duplicates (removed in second screening) were intentionally skewed to reduce the number of apps assigned to each Google Play reviewer. Prior to removal, content of app was checked between-stores to ensure both (App Store & Google Play) versions displayed identical content.
