# Supplementary material for: Evaluating Evidence-Based Content, Features of Exercise Instruction, and Expert Involvement in Physical Activity Apps for Pregnant Women: Systematic Search and Content Analysis
Source: JMIR Mhealth Uhealth. 2022 Jan 19;10(1):e31607. doi: 10.2196/31607 (PMC8811692; doi:10.2196/31607)
Supplement: Multimedia Appendix 2 [file mhealth_v10i1e31607_app2.docx]

**Multimedia Appendix 2: Data Extraction Instructions and Extraction Tool**

*Data Extraction Instructions*

- Ensure you have reviewed the Data Extraction Tool, so you are familiar with the data you are looking to extract.
  (Some information of interest, such as requested profile information, terms and conditions, and/or disclaimers may be presented at this early stage. If so, it may be a good idea to take a screen shot or complete those sections in the data extraction tool.)
- Download the app to be reviewed, accepting any free trial version of the app.
- Use the following information and user profiles to complete the data extraction tool.

*
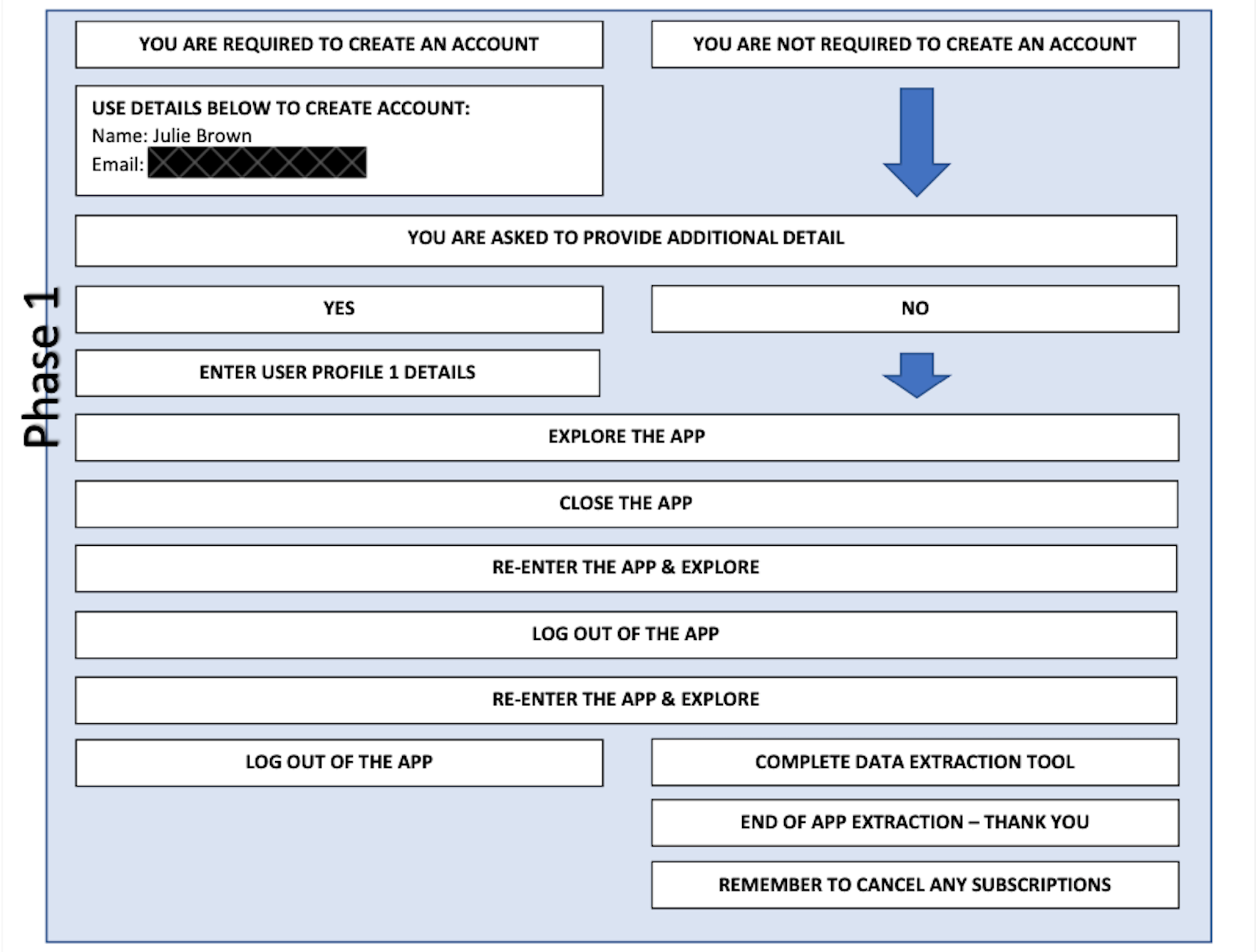
*

**USER PROFILE 1: Julie Smith 1**

- Name: Julie Smith
- Age: 28
- Gender: Female
- Height: 175 cm / 5.7 feet
- Current Weight: 87 kg / 192 pounds
- Weight prior to current pregnancy: 70 kg / 154 pounds
- Email: [REDACTED]
- Phone: [REDACTED]
- Country: [REDACTED]
- Zip Code / Postcode: [REDACTED]

**Medical history / Contraindications to exercise during pregnancy:**

- Currently pregnant with twins over 28 weeks gestation
- No other conditions or contraindications

**Information regarding current pregnancy:**

- Estimated due date: 8^th^ January 2021
- Current trimester: 3
- Gender of babies: Make it up
- No of babies: 2
- This is my first pregnancy

**Current exercise behaviours:**

- Frequency: On most, if not all days of the week
- Intensity: Moderate – vigorous intensity exercise
- Time: Engaged in 150 – 300 minutes per week
- Type: Strength training x 2, running and pregnancy specific aerobic classes per week

**Exercise intentions:**

- Frequency: On most, if not all days of the week
- Intensity: Moderate intensity exercise
- Time: Engaged in at least 150 minutes per week
- Type: Strength training x 2 and pregnancy specific aerobic classes per week
- Goal: To stay fit and active

**IMPORTANT:**

Once you have worked your way through the information within the app using this profile, go back and make changes to the information you provided (using the details from User Profile 2 below) to see if any of the app content changes / updates. You will need to do this to complete the data extraction tool. It is important we know whether an app adjusts / modifies its content based on the information that you provide.

Alternatively, you might prefer to make changes to the user information you provide as you progress throughout the app. This approach is perfectly fine also. Chose the approach that suits you best.

*
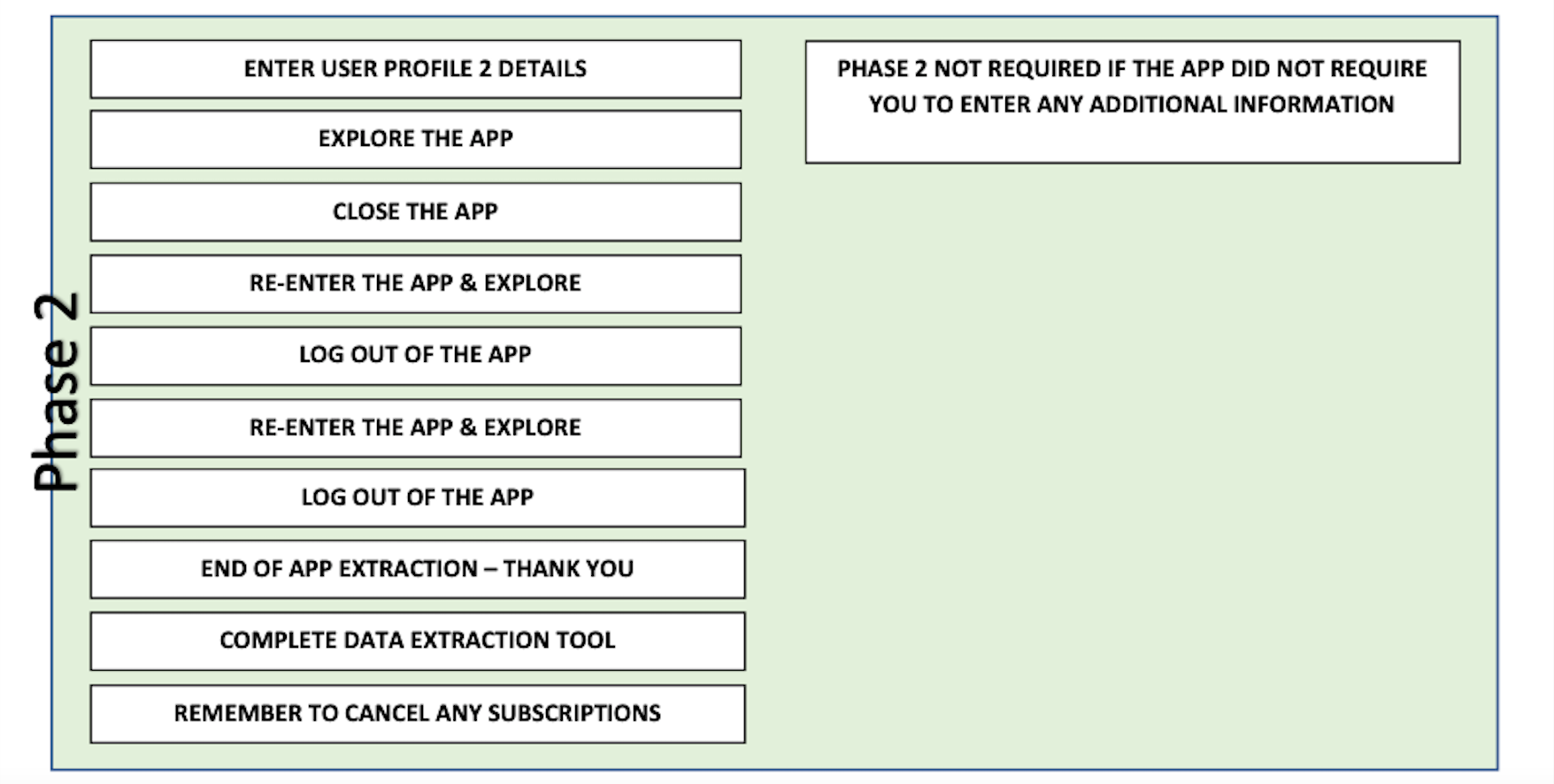
***USER PROFILE 2: Julie Smith 2**

- Age: 42
- Height: 160 cm / 5.2 feet
- Current Weight: 80 kg / 176 pounds
- Weight prior to current pregnancy: 70 kg / 154 pounds

**Medical history / Contraindication to exercise during pregnancy:**

- No conditions or contraindications

**Information regarding current pregnancy:**

- Estimated due date: 25^th^ December 2020
- Current trimester: 2
- Gender of babies: unknown
- No of babies: 1
- This is my third pregnancy

**Current exercise behaviours:**

- Frequency: 2-3 days a week
- Intensity: Light - moderate intensity exercise
- Time: 20-30 minutes per sessions
- Type: walking

**Exercise intentions:**

- Frequency: On most, if not all days of the week
- Intensity: Moderate intensity exercise
- Time: Engaged in at least 150 minutes per week
- Type: Strength training x 2 and pregnancy specific aerobic classes per week
- Goal: Improve fitness for mine and my baby’s health

*Data Extraction Tool*

| **Reviewer Details** | | | |
| --- | --- | --- | --- |
| Q1. | Reviewer Name | | |
| Q2. | Name of the App   - Baby2Body: Pregnancy Wellness - Emily Skye FIT: Workout App - Juna - Pregnancy Workouts - Moms Into Fitness - Pregnancy + - Prenatal Yoga \| Down Dog - Tips for Pregnant: Hello Belly - Tone It Up: Workout & Fitness - U Pilates: Workouts & Exercise - Yoggy: pregnancy yoga workouts - YogiBirth: Pregnancy Yoga App - 9MonthsGuide - Happy Pregnancy App - Healthy Pregnancy Tips - I'm Pregnant - Pregnancy Week By Week - Jillian Michaels: The Fitness App - Kegel Exercises for Men & Women - A How-to Guide - Move Your Bump - My pregnancy calendar app: baby countdown timer - My Pregnancy Journey - pregnancy calendar - Pregnancy Companion - Week by Week Tracking - Pregnancy Exercise and workout at home Pregnancy Exercises - Pregnancy Guide - Pregnancy Guide App - Pregnant. Pregnancy by week. Pregnancy calendar | | |
| **User Details** | | | |
| Q3. | Does the app require the user to create an account before allowing access to any content?   - Yes - No | | |
| Q4. | Does the app ask the user for any of the following personal information? (*Select all that apply*)   - Name - Age - Date of Birth (DOB) - Height - Current Weight - Weight prior to current pregnancy - Country - Other personal information OR additional information regarding your selections? Please detail below:  _______________________________________________________________________________________ - No, the app does not ask the user to provide any personal information | | |
| **App Screening Questions** | | | |
| Q5. | Does the app have a Terms and Conditions or Disclaimer?   - Yes - No | | |
|  | IF YES → | Does the app require the user to agree to Terms and Conditions?   - Yes - No | |
|  |  | Does the app require the user to agree to a Disclaimer?   - Yes - No | |
|  |  | Do the Terms and Conditions and/or Disclaimer state anything about the following? (*Select all that apply*)   - User participation is at own risk - The app is not responsible for any adverse outcomes that may occur when using the app, or as a result of the app - It is the user’s responsibility to seek clearance from a health professional before using the app - It is the users responsible to ensuring the app is safe for them to use - The user is responsible for making any modifications / adjustments to exercise whilst using the app - Other information specific to exercise OR additional information regarding your selections? Please detail below:  _______________________________________________________________________________ | |
| Q6. | Does the app specifically ask the user if they have approval / clearance from a qualified professional (e.g., health professional, exercise professional) to engage with the app, prior to giving the user access to exercise content?   - Yes - No | | |
| **Health Screening** | | | |
| Q7. | Does the app ask the user if they have ever experienced any of the following conditions prior to pregnancy? (S*elect all that apply*)   - Heart condition or stroke - Unexplained pains or discomfort in the chest - Faint, dizzy or lose of balance during exercise - Asthma attack requiring immediate medical attention in last 12 months - Diabetes and had trouble controlling blood sugar (glucose) in last 3 months - Muscle bone or joint problems that may be made worse by participating in exercise - Other condition/s that may require special consideration to exercise OR additional information regarding your selections? Please detail below:  _______________________________________________________________________________________ - No, the app does not ask the user if they have experienced any conditions prior to pregnancy | | |
| Q8. | Does the app ask the user if they have ever experienced any of the following contraindications to exercise during pregnancy? (*Select all that apply*)   - Incompetent cervix - Ruptured membranes, premature labour - Persistent second or third trimester bleeding - Placenta previa - Pre-eclampsia - Evidence of intrauterine growth restriction - Multiple gestation (e.g., triplets or higher number) - Poorly controlled Type I diabetes, hypertension, or thyroid disease - Other serious cardiovascular, respiratory, or systemic disorder - History of spontaneous miscarriage, premature labour, or fetal growth restriction - Mild/moderate cardiovascular or chronic respiratory disease - Pregnancy-induced hypertension - Poorly controlled seizure disorder - Type 1 diabetes - Symptomatic anaemia - Malnutrition, significantly underweight or eating disorder - Twin pregnancy after the 28th week - Other contraindications to exercise during pregnancy OR additional information regarding your selections? Please detail below:  _______________________________________________________________________________________ - No, the app does not ask the user if they have experienced any contraindications to exercise during pregnancy | | |
| Q9. | Does the app ask the user for any of the following information regarding their current pregnancy? (*Select all that apply*)   - Estimated due date - Current Trimester - How many babies are expected (singleton / twins / triplets etc.) - What number pregnancy this is (first, second, third pregnancy etc.) - Other information regarding the current pregnancy OR additional information regarding your selections? Please detail below:  _______________________________________________________________________________________ - No, the app does not ask the user to provide any of information regarding their current pregnancy | | |
| **User Exercise Behaviours** | | | |
| Q10. | Does the app ask the user any questions about their CURRENT EXERCISE BEHAVIOURS?   - Yes - No | | |
|  | IF YES → | Which of the following current exercise behaviours does the app ask about? (*Select all that apply*)   - Frequency (How often the user exercises per week) - Intensity (What intensity the user exercises at) - Time (How many total minutes the user exercises each week or how many minutes each exercise session is) - Type (What types of exercise the user engages in) - Other exercise behaviours OR additional information regarding your selections? Please detail below:  _______________________________________________________________________________ | |
| **User Exercise Intentions** | | | |
| Q11. | Does the app ask the user any questions about their CURRENT EXERCISE INTENTIONS?   - Yes - No | | |
|  | IF YES → | Which of the following exercise intentions does the app ask about? (*Select all that apply*)   - Frequency (How often the user wants to exercise per week) - Intensity (What intensity the user wants the exercise to be) - Time (Total minutes the user wants to exercise each week or how many minutes the user wants each exercise session to be) - Type (What types of exercise the user wants to engage in) - Goal for using the app (get active/get fit/stay fit/get strong/manage weight)? - Other exercise intentions? Please detail below:  _______________________________________________________________________________ | |
| **User Information Updates** | | | |
| Q12. | When closing and then reopening the app, is the user asked if any of their following details have changed / need to be updated? (*Select all that apply*)   - Personal Details (e.g. height, weight) - Medical and/or pregnancy conditions (e.g. feeling faint or dizzy when exercising) - Exercise behaviours (e.g., only doing 2 sessions a week instead of 4) - Exercise intentions (e.g., no longer want to engage in strength training) - Other details that have changed / need to be updated OR additional information regarding your selections? Please detail below:  _______________________________________________________________________________________ - No, the app does not ask the user if their details have changed or need to be updated when reopening the app | | |
| Q13. | When logging out of the app and then logging back into the app again, is the user asked if any of their following details have changed / need to be updated? (*Select all that apply*)   - Personal Details (e.g., height, weight) - Medical and/or pregnancy conditions (e.g., feeling faint or dizzy when exercising) - Exercise behaviours (e.g., only doing 2 sessions a week instead of 4) - Exercise intentions (e.g., no longer want to engage in strength training) - Other details that have changed / need to be updated OR additional information regarding your selections? Please detail below:  _______________________________________________________________________________________ - No, the app does not ask the user if their details have changed or need to be updated when logging back into the app | | |
| Q14. | Which of the following statements best describes the app:   - Pregnancy-specific exercise app - General pregnancy app, that also provides exercise-specific content - Generic exercise app, that also provides pregnancy-specific exercise content | | |
| Q15. | Which of the following statements best describes the app:   - An app consisting of different types of exercise (e.g., a pregnancy app that offers a variety of aerobic, strength, pelvic floor, flexibility exercises / workouts) - An app consisting of only one type of exercise (e.g., a yoga app that only offers a variety of yoga exercises / workouts) | | |
| Q16. | Please describe the main exercise features of the app (without in-app purchases).  *For example: This app provides users with 1 x 3-minute exercise video each week. Each video consists of the instructor demonstrating and performing one exercise, which is a breathing or joint mobility (mostly pelvic area) exercise. Access to each video is limited and only become available when your gestation increases. E.g. - I can only access week 7 exercise video once I am 7 weeks pregnant and access to past videos disappears as gestation increases*  *For example: This app offers unlimited access to a library of 30 individual exercises, target all major muscle groups, which are mostly body weight exercises. In some instances, you need small hand-held weights/or similar to perform an exercise. There is a video demonstrating each exercise. Each video lasts approximately 30 seconds.*  ______________________________________________________________________________________________  ______________________________________________________________________________________________  ______________________________________________________________________________________________  ______________________________________________________________________________________________ | | |
| Q17. | What type/s of exercises / workouts does the app provide? (*Select all that apply*)   - Yoga - Stretching - Pilates - Flexibility - High Intensity Interval Training (HIIT) - Pelvic Floor / Kegel exercises - Walking / Jogging / Running - Cycling - Swimming - Resistance Training (body weight, resistance bands) - Weight Training (must include weights) - Other exercises OR additional information regarding your selections? Please detail below:  _______________________________________________________________________________________ | | |
| Q18. | Which of the following does the app provide? (*Select all that apply*)   - The app provides a library of individual exercises - The app provides a library of individual workouts (each workout contains a series of pre-determined exercises) - The app provides a library of programs (each program contains a series of pre-determined workouts) - None of the above. Please detail below:  _______________________________________________________________________________________ | | |
| Q19. | Which of the following features does the app use to demonstrate and/or explain an exercise? (*Select all that apply*)   - Spoken cues - Written cues - Still images / pictures demonstrating an exercise - Video/s demonstrating an exercise | | |
| Q20. | Does the app provide any of the following information upfront to help inform the user's choice of exercise / workout? (*Select all that apply*)   - Frequency of exercise (e.g., workout will include 3 rounds, where user performs 10-12 repetitions per exercise per round) - Duration of exercise session (e.g., workout will go for 20 minutes) - Intensity of exercise session (e.g., workout will include short bouts of high intensity exercise) - Type of exercise session (e.g., workout will target lower body or workout is designed to increase heart rate) - Experience Level required (e.g., workout is designed for beginners) - Equipment required (e.g., a small set of dumbbells are required for this workout) - Trimester (e.g., workout is designed for the second trimester) - Other information to help inform the user's choice of exercise / workout OR additional information regarding your selections? Please detail below:  _______________________________________________________________________________________ - No, the app does not provide any upfront information to help inform the users choice of exercise | | |
| Q21. | Does the app allow the user to select / modify any of the exercise / workout characteristics? (e.g., frequency, intensity, duration, type)  *E.g., The app allows the user to select/modify how long they want a particular Yoga workout to be, or to select the intensity in which the user wishes to exercise at.*   - Yes - No | | |
|  | IF YES → | Which of the following exercise / workout characteristics does the app allow the user to select / modify? (*Select all that apply*)   - Frequency of the exercise / workout - Intensity of the exercise / workout - Duration of the exercise / workout - Type of the exercise / workout | |
|  |  | Does the app provide any feedback / recommendations to the user about the selections / modifications they make to the exercise / workout characteristics?  *E.g., The user tells the app that they want to complete 10 x 45-minute high-intensity exercise sessions/workouts a week. Does the app provide the user with feedback advising them that they may be exceeding the recommended amount of exercise per week?*   - Yes - No | |
|  |  | IF YES → | If the user selects / modifies the exercise / workout characteristics within the app, what feedback / recommendations does the app provide to the user? (*Select all that apply*)   - Exceeding recommended amount of exercise Meeting recommended amount of exercise - Not meeting recommended amount of exercise - User selections / modifications are not recommended User selections / modifications are recommended - User should proceed under the supervision of a qualified professional - Other feedback / recommendations OR additional information regarding your selections? Please detail below:  ______________________________________________________________________ |
| **Additional App Exercise Content** | | | |
| Q22. | Does the app provide any information on the benefits of exercise during pregnancy? (*Select all that apply*)   - Maintains or improves cardiorespiratory fitness - Reduces risk of pregnancy conditions including gestational diabetes, pre-eclampsia, and hypertension - Reduces risk of obstetric intervention (C-section, instrumental delivery) - Improves mental health - Reduces physical discomforts - Assists with postpartum recovery - Reduces the risk of urinary incontinence - Other information regarding the benefits of exercise during pregnancy OR additional information regarding your selections? Please detail below:  _______________________________________________________________________________________ - No, the app does not provide any information on the benefits of exercise during pregnancy | | |
|  | IF YES → | Where is the information on the benefits of exercise during pregnancy located? (*Select all that apply*)   - App Homepage - Disclaimer and/or Terms and Conditions - Information tab / Information section within the app - Within the exercise instructions, demonstrations, workout videos - Another location OR additional information regarding your selections? Please detail below:  _______________________________________________________________________________ | |
| Q23. | Does the app provide any information on the contraindications to exercise during pregnancy? (*Select all that apply*)   - Incompetent cervix - Ruptured membranes, premature labour - Persistent second or third trimester bleeding - Placenta previa - Pre-eclampsia - Evidence of intrauterine growth restriction - Multiple gestation (e.g., triplets or higher number) - Poorly controlled Type I diabetes, hypertension, or thyroid disease - Other serious cardiovascular, respiratory, or systemic disorder - History of spontaneous miscarriage, premature labour, or fetal growth restriction - Mild/moderate cardiovascular or chronic respiratory disease - Pregnancy-induced hypertension - Poorly controlled seizure disorder - Type 1 diabetes - Symptomatic anaemia - Malnutrition, significantly underweight or eating disorder - Twin pregnancy after the 28th week - Other information regarding contraindication to exercise during pregnancy OR additional information regarding your selections? Please detail below:  _______________________________________________________________________________________ - No, the app does not provide any information on the contraindications to exercise during pregnancy | | |
|  | IF YES → | Where is the information on the contraindications to exercise during pregnancy located? (*Select all that apply*)   - App Homepage - Disclaimer and/or Terms and Conditions - Information tab / Information section within the app - Within the exercise instructions, demonstrations, workout videos - Another location OR additional information regarding your selections? Please detail below:  _______________________________________________________________________________ | |
| Q24. | Does the app provide any information on the signs and symptoms to cease exercise during pregnancy? (*Select all that apply*)   - Chest pain - Persistent excessive shortness of breath - that does not resolve with rest - Severe headache - Persistent dizziness/feeling faint - that does not resolve with rest - Regular painful uterine contractions - Vaginal bleeding - Persistent loss of fluid from the vagina - indicating possible ruptured membrane - Other information regarding signs/symptoms to cease exercise during pregnancy OR additional information regarding your selections? Please detail below:  _______________________________________________________________________________________ - No, the app does not provide any information on the signs and symptoms to cease exercise during pregnancy | | |
|  | IF YES → | Where is the information on the signs and symptoms to cease exercise during pregnancy located? (*Select all that apply*)   - App Homepage - Disclaimer and/or Terms and Conditions - Information tab / Information section within the app - Within the exercise instructions, demonstrations, workout videos - Another location OR additional information regarding your selections? Please detail below: _______________________________________________________________________________ | |
| Q25. | Does the app provide any information on the frequency of exercise during pregnancy? (*Select all that apply*)   - Exercise should be performed on most, if not all days of the week - Women should engage in 2 sessions of resistance-based exercise per week - Other information regarding frequency of exercise during pregnancy OR additional information regarding your selections? Please detail below:  _______________________________________________________________________________________ - No, the app does not provide any information on the frequency of exercise during pregnancy | | |
|  | IF YES → | Where is the information on the frequency of exercise during pregnancy located? (*Select all that apply*)   - App Homepage - Disclaimer and/or Terms and Conditions - Information tab / Information section within the app - Within the exercise instructions, demonstrations, workout videos - Another location OR additional information regarding your selections? Please detail below:  _______________________________________________________________________________ | |
| Q26. | Does the app provide any information on the intensity of exercise during pregnancy? (*Select all that apply*)   - Light intensity - Moderate intensity - Vigorous intensity - Use the Borg Rating of Perceived Exertion (RPE) scale to judge the intensity of exercise - Using the RPE (scale 1 - 20), RPE range of 11-17 is considered safe - Use Talk Test to judge intensity of exercise - Using the Talk Test, women should be able to carry on a conversation or sing, while in vigorous exercise they would find this difficult to do - Use Heart Rate zones (based on age and fitness level) to judge the intensity of exercise - Other information regarding intensity of exercise during pregnancy OR additional information regarding your selections? Please detail below:  _______________________________________________________________________________________ - No, the app does not provide any information on the intensity of exercise during pregnancy | | |
|  | IF YES → | Where is the information on the intensity of exercise during pregnancy located? (*Select all that apply*)   - App Homepage - Disclaimer and/or Terms and Conditions - Information tab / Information section within the app - Within the exercise instructions, demonstrations, workout videos - Another location OR additional information regarding your selections? Please detail below:  _______________________________________________________________________________ | |
| Q27. | Does the app provide any information on the duration of exercise during pregnancy? (*Select all that apply*)   - Accumulate between 150 - 300 minutes of exercise per week - Accumulate at least 150 minutes of exercise per week - Exercise for 30 minutes per day - Exercise for at least 15 minutes per session - Break up exercise into small bouts - Other information regarding duration of exercise during pregnancy OR additional information regarding your selections? Please detail below:  _______________________________________________________________________________________ - No, the app does not provide any information on the duration of exercise during pregnancy | | |
|  | IF YES → | Where is the information on the duration of exercise during pregnancy located? (*Select all that apply*)   - App Homepage - Disclaimer and/or Terms and Conditions - Information tab / Information section within the app - Within the exercise instructions, demonstrations, workout videos - Another location OR additional information regarding your selections? Please detail below:  _______________________________________________________________________________ | |
| Q28. | Does the app provide any information on the type of exercise that is safe during pregnancy? (*Select all that apply*)   - Aerobic physical activity / exercise (e.g., brisk walking, cycling, swimming, dancing, exercise classes) - Muscle strengthening exercises using body weight, weights, or resistance bands - Pelvic floor muscle exercises - Pregnancy specific classes - Other information regarding exercises considered safe during pregnancy OR additional information regarding your selections? Please detail below:  _______________________________________________________________________________________ - No, the app does not provide any information on the type of exercise that is safe during pregnancy | | |
|  | IF YES → | Where is the information on the type of exercise that is safe during pregnancy located? (*Select all that apply*)   - App Homepage - Disclaimer and/or Terms and Conditions - Information tab / Information section within the app - Within the exercise instructions, demonstrations, workout videos - Another location OR additional information regarding your selections? Please detail below:  _______________________________________________________________________________ | |
| Q29. | Does the app provide any information on the type of exercise that is not safe during pregnancy? (*Select all that apply*)   - Significant changes in pressure (e.g., sky diving, scuba diving) - Risk of contact / collision - Risk of falling (e.g., exercise that requires high levels of balance, coordination, and agility) - Heavy lifting - Other information regarding exercises not considered safe during pregnancy OR additional information regarding your selections? Please detail below:  _______________________________________________________________________________________ - No, the app does not provide any information on the type of exercise that is unsafe during pregnancy | | |
|  | IF YES → | Where is the information on type of exercise that is not safe during pregnancy located? (*Select all that apply*)   - App Homepage - Disclaimer and/or Terms and Conditions - Information tab / Information section within the app - Within the exercise instructions, demonstrations, workout videos - Another location OR additional information regarding your selections? Please detail below:  _______________________________________________________________________________ | |
| Q30. | Does the app provide any additional information / recommendations on exercise during pregnancy? (*Select all that apply*)   - Avoid dehydration - Avoid inadequate nutrition - Stay well hydrated - Try to ensure energy intake is in line with recommended gestational weight gain - Avoid heat stress/hyperthermia in the first trimester - Adjust physical activity / exercise in excessively hot weather or high humidity - Avoid long periods of standing still - Avoid long periods of laying in the supine position - Avoid physical activity / exercise at high altitude - Always wear appropriate clothing (shoes for the activity, non-restrictive clothing and a supportive bra) - If sedentary or inactive, commence exercise slowly and progress toward meeting the recommended guidelines - Women who are healthy and already active do not need to seek medical clearance for physical activity / exercise during pregnancy - Minimise the amount of time spent in prolonged sitting - Break up long periods of sitting as often as possible - As pregnancy progresses, anatomical and physiological/metabolic changes mean that modifications to some activities/exercises are required. - Women who are considering high volumes of exercise training (high intensity, prolonged duration, heavy weights, etc.) should seek advice and guidance from a health professional who is knowledgeable about the effects of high-level training on maternal and fetal outcomes - Women should seek clearance before commencing exercise during pregnancy - Other information / recommendations OR additional information regarding your selections? Please detail below:  _______________________________________________________________________________________ - No, the app does not provide any other information regarding exercise during pregnancy | | |
|  | IF YES → | Where is the information on additional information / recommendations during pregnancy located? (*Select all that apply*)   - App Homepage - Disclaimer and/or Terms and Conditions - Information tab / Information section within the app - Within the exercise instructions, demonstrations, workout videos - Another location OR additional information regarding your selections? Please detail below:  _______________________________________________________________________________ | |
| **App Developer Qualifications, Credibility, and Expertise** | | | |
| Q31. | Does the app provide a list of developer qualifications?  *E.g., Personal Trainer, Exercise Professional, University degree, CrossFit coach, Advanced Yoga instructor*   - Yes - No | | |
|  | IF YES → | Please list these qualifications in the text box below:  ______________________________________________________________________________________  ______________________________________________________________________________________  ______________________________________________________________________________________  ______________________________________________________________________________________ | |
| Q32. | Does the app provide any information to imply developer credibility and/or expertise, that is not a recognised qualification?  E.g., The app developer has now had two healthy babies so knows this and that, or the app developer has been a fitness trainer for the past 6 years, the app developer has teamed up with women’s health experts to bring you this app, the app developer is a CrossFit athlete who has trained throughout two pregnancies now and has two healthy children  Yes  No | | |
|  | IF YES → | Please list the information that has been provided to imply app developer credibility and/or expertise in the space below:  ______________________________________________________________________________________  ______________________________________________________________________________________  ______________________________________________________________________________________  ______________________________________________________________________________________ | |
| Q33. | Does the app reference any of the following sources of information? (*Select all that apply*)   - Academic Literature - Government Guidelines (exercise guidelines that incorporate pregnancy and/or exercise during pregnancy guidelines - OBGYN Guidelines - Industry Body Guidelines - Other sources of information OR additional information regarding your selections? Please detail below:  _______________________________________________________________________________________ - No, the app does not reference any sources of information | | |
| Q34. | Does the app provide a reference list?   - Yes - No | | |
| **Additional Feedback** | | | |
| Q35. | Please provide any additional comments / information that you have regarding this app that was not collected as a part of the extraction process.  ______________________________________________________________________________________________  ______________________________________________________________________________________________  ______________________________________________________________________________________________  ______________________________________________________________________________________________  ______________________________________________________________________________________________ | | |
