# Supplementary material for: Evaluating Evidence-Based Content, Features of Exercise Instruction, and Expert Involvement in Physical Activity Apps for Pregnant Women: Systematic Search and Content Analysis
Source: JMIR Mhealth Uhealth. 2022 Jan 19;10(1):e31607. doi: 10.2196/31607 (PMC8811692; doi:10.2196/31607)
Supplement: Multimedia Appendix 3 [file mhealth_v10i1e31607_app3.docx]

**Multimedia Appendix 3: Personal Information, Terms and Conditions, and Disclaimer**

*Apps asking personal and pregnancy related information and providing terms and conditions or disclaimers (detailed).*

| App ID | | 01 | 02 | 03 | 04 | 05 | 06 | 07 | 08 | 09 | 10 | 11 | 12 | 13 | 14 | 15 | 16 | 17 | 18 | 19 | 20 | 21 | 22 | 23 | 24 | 25 | 26 | 27 | *n* |
| --- | --- | --- | --- | --- | --- | --- | --- | --- | --- | --- | --- | --- | --- | --- | --- | --- | --- | --- | --- | --- | --- | --- | --- | --- | --- | --- | --- | --- | --- |
| Requires user account | |  | X |  |  | X |  | X |  | X |  | X |  |  |  | X |  | X | X | X | X | X | X |  | X | X |  | X | 15 |
| Personal information | |  |  |  |  |  |  |  |  |  |  |  |  |  |  |  |  |  |  |  |  |  |  |  |  |  |  |  |  |
|  | Name | X | X |  |  | X |  | X |  | X |  | X |  |  |  | X |  | X | X | X | X | X |  |  | X | X |  | X | 15 |
|  | Contact details (email / phone / other) |  | X |  |  |  |  | X |  |  |  | X |  |  |  |  |  | X | X | X | X | X |  |  |  |  |  |  | 8 |
|  | Current weight | X |  |  | X | X |  |  |  | X | X |  |  |  |  |  |  | X |  |  |  | X |  |  |  |  |  |  | 7 |
|  | Date of birth / Age |  | X |  |  | X |  |  |  | X |  |  |  |  |  |  |  |  |  | X |  | X |  |  |  | X |  | X | 7 |
|  | Height |  |  |  | X | X |  |  |  |  | X | X |  |  | X |  |  |  |  |  |  |  |  |  |  |  |  |  | 5 |
|  | Country / Location |  | X |  |  |  |  |  |  | X |  |  |  |  |  |  |  |  | X |  |  |  |  |  | X |  |  |  | 4 |
|  | Gender |  | X |  |  | X |  |  |  |  |  |  |  |  |  |  |  |  | X |  |  |  |  |  |  | X |  |  | 4 |
| Information on current pregnancy | |  |  |  |  |  |  |  |  |  |  |  |  |  |  |  |  |  |  |  |  |  |  |  |  |  |  |  |  |
|  | Gestation (due date, last menstrual period, date of conception, current trimester, etc) | X | X |  | X |  |  |  | X | X | X | X |  |  | X | X |  | X |  | X |  | X |  | X |  |  |  | X | 14 |
|  | Number of babies expected (singleton, twins, triplets, etc) |  |  |  |  |  |  |  |  | X |  |  |  |  |  |  |  | X |  |  |  | X |  |  |  |  |  | X | 4 |
|  | Number pregnancy (first, second, third pregnancy) |  |  |  |  |  |  |  |  |  |  |  |  |  |  |  |  | X |  |  |  | X |  |  |  |  |  | X | 3 |
|  | Weight prior to pregnancy |  |  |  | X |  |  |  |  |  | X | X |  |  |  |  |  |  |  |  |  |  |  |  |  |  |  |  | 3 |
|  | Relationship to baby (Mother, Father, Grandparent, etc) |  |  |  |  |  |  |  |  | X |  |  |  |  |  |  |  |  |  |  |  | X |  |  |  |  |  |  | 2 |
|  | Baby’s gender |  |  |  |  |  |  |  |  | X |  |  |  |  |  |  |  |  |  |  |  | X |  |  |  |  |  |  | 2 |
| Terms & Conditions / Disclaimer | |  |  |  |  |  |  |  |  |  |  |  |  |  |  |  |  |  |  |  |  |  |  |  |  |  |  |  |  |
|  | Presents Terms & Conditions and/or Disclaimer | X | X |  |  | X | X |  |  | X |  | X | X |  | X | X |  | X | X | X | X | X | X |  | X | X | X | X | 19 |
|  | Recommended that women should seek medical clearance before commencing exercise during pregnancy | X |  | X |  | X |  |  | X | X | X | X | X |  | X |  | X |  | X | X | X | X |  | X |  | X | X | X | 18 |
|  | Required Terms & Conditions agreement |  |  |  |  | X |  |  |  | X |  |  | X |  | X | X |  |  | X | X |  | X |  |  |  | X |  | X | 10 |
|  | Required Disclaimer agreement |  |  |  |  | X |  |  |  |  |  |  |  |  |  |  |  | X | X | X |  |  | X |  |  |  |  |  | 5 |
