# Supplementary material for: Evaluating Evidence-Based Content, Features of Exercise Instruction, and Expert Involvement in Physical Activity Apps for Pregnant Women: Systematic Search and Content Analysis
Source: JMIR Mhealth Uhealth. 2022 Jan 19;10(1):e31607. doi: 10.2196/31607 (PMC8811692; doi:10.2196/31607)
Supplement: Multimedia Appendix 4 [file mhealth_v10i1e31607_app4.docx]

**Multimedia Appendix 4. Contraindications and behavior and intention screening.**

*Information about contraindications (detailed).*

| App Identifier | | | 01 | 02 | 03 | 04 | 05 | 06 | 07 | 08 | 09 | 10 | 11 | 12 | 13 | 14 | 15 | 16 | 17 | 18 | 19 | 20 | 21 | 22 | 23 | 24 | 25 | 26 | 27 | *n* |
| --- | --- | --- | --- | --- | --- | --- | --- | --- | --- | --- | --- | --- | --- | --- | --- | --- | --- | --- | --- | --- | --- | --- | --- | --- | --- | --- | --- | --- | --- | --- |
| Information about contraindications | | |  |  |  |  |  |  |  |  |  |  |  |  |  |  |  |  |  |  |  |  |  |  |  |  |  |  |  |  |
|  | History of spontaneous miscarriage, premature labour or fetal growth restriction | | X | X |  |  |  |  |  | X | X | X |  |  |  |  |  | X |  |  | X |  | X |  |  |  |  |  |  | 8 |
|  | Placenta previa | | X | X |  |  |  |  |  | X | X |  |  |  |  |  |  | X |  |  | X |  | X |  |  |  |  |  |  | 7 |
|  | Persistent second or third trimester (vaginal/uterine) bleeding | |  | X |  |  |  |  |  |  | X | X |  |  |  | X |  | X |  |  | X |  | X |  |  |  |  |  |  | 7 |
|  | Serious cardiovascular, respiratory or systemic disorder | | X | X |  |  |  |  |  |  | X |  |  |  |  |  |  | X |  |  |  |  | X |  |  |  |  |  |  | 5 |
|  | Incompetent cervix | | X |  |  |  |  |  |  |  | X | X |  |  |  |  |  |  |  |  | X |  | X |  |  |  |  |  |  | 5 |
|  | Ruptured membranes, premature labour | |  |  |  |  |  |  |  |  | X | X |  |  |  | X |  | X |  |  | X |  |  |  |  |  |  |  |  | 5 |
|  | Pre-eclampsia | |  |  |  |  |  |  |  |  | X |  |  |  |  | X |  | X |  |  | X |  | X |  |  |  |  |  |  | 5 |
|  | Mild / moderate cardiovascular or chronic respiratory disease | | X |  |  |  |  |  |  |  | X |  |  |  |  |  |  | X |  |  |  |  | X |  |  |  |  |  |  | 4 |
|  | Symptomatic anaemia | |  |  |  |  |  |  |  |  | X |  |  |  |  |  |  | X |  |  |  |  | X |  |  |  |  |  |  | 3 |
|  | Evidence of intrauterine growth restriction | |  |  |  |  |  |  |  |  | X | X |  |  |  |  |  |  |  |  |  |  |  |  |  |  |  |  |  | 2 |
|  | Multiple gestation (e.g., triplets or higher number) | |  |  |  |  |  |  |  |  | X |  |  |  |  |  |  | X |  |  |  |  |  |  |  |  |  |  |  | 2 |
|  | Pregnancy-induced hypertension | |  |  |  |  |  |  |  |  |  | X |  |  |  |  |  |  |  |  |  |  | X |  |  |  |  |  |  | 2 |
|  | Poorly controlled type I diabetes, hypertension or thyroid disease | | X |  |  |  |  |  |  |  |  |  |  |  |  |  |  |  |  |  |  |  |  |  |  |  |  |  |  | 1 |
|  | Twin pregnancy after the 28^th^ week | |  |  |  |  |  |  |  |  |  |  |  |  |  |  |  |  |  |  |  | X |  |  |  |  |  |  |  | 1 |
|  | Other | |  |  |  |  |  |  |  |  |  |  |  |  |  |  |  |  |  |  |  |  |  |  |  |  |  |  |  |  |
|  |  | Chronic health issues (non-specified) |  |  |  |  |  |  | X |  |  |  |  |  |  |  |  |  |  |  |  |  |  |  |  |  |  |  |  | 1 |
|  |  | Pregnancy related complications (non-specified) |  |  |  |  |  |  | X |  |  |  |  |  |  |  |  |  |  |  |  |  |  |  |  |  |  |  |  | 1 |
|  |  | High body weight |  |  |  |  |  |  |  | X |  |  |  |  |  |  |  |  |  |  |  |  |  |  |  |  |  |  |  | 1 |
|  |  | Infectious disease |  |  |  |  |  |  |  |  |  |  |  |  |  |  |  | X |  |  |  |  |  |  |  |  |  |  |  | 1 |
|  |  | Inflammatory disease |  |  |  |  |  |  |  |  |  |  |  |  |  |  |  | X |  |  |  |  |  |  |  |  |  |  |  | 1 |
|  |  | Chronic toxicities requiring hospitalisation |  |  |  |  |  |  |  |  |  |  |  |  |  |  |  | X |  |  |  |  |  |  |  |  |  |  |  | 1 |
|  |  | Low abdominal pain |  |  |  |  |  |  |  |  |  |  |  |  |  |  |  | X |  |  |  |  |  |  |  |  |  |  |  | 1 |
|  |  | High blood pressure |  |  |  |  |  |  |  |  |  |  |  |  |  |  |  |  |  |  |  |  | X |  |  |  |  |  |  | 1 |

*Screening of exercise behaviour, intentions and preferences (detailed).*

| App Identifier | | 01 | 02 | 03 | 04 | 05 | 06 | 07 | 08 | 09 | 10 | 11 | 12 | 13 | 14 | 15 | 16 | 17 | 18 | 19 | 20 | 21 | 22 | 23 | 24 | 25 | 26 | 27 | *n* |
| --- | --- | --- | --- | --- | --- | --- | --- | --- | --- | --- | --- | --- | --- | --- | --- | --- | --- | --- | --- | --- | --- | --- | --- | --- | --- | --- | --- | --- | --- |
| Current PA/exercise behaviour | |  |  |  |  |  |  |  |  |  |  |  |  |  |  |  |  |  |  |  |  |  |  |  |  |  |  |  |  |
|  | Frequency (how often the user exercises per week) |  |  |  |  |  |  |  |  |  |  |  |  |  |  |  |  | X |  |  |  |  |  |  |  |  |  |  | 1 |
|  | Intensity (what intensity the user exercises at) |  |  |  |  | X |  |  |  |  |  |  |  |  |  |  |  |  |  |  |  |  |  |  |  | X |  |  | 2 |
|  | Type (what types of exercise the user engages in) |  |  |  |  |  |  |  |  |  |  |  |  |  |  |  |  |  |  |  |  |  |  |  | X |  |  |  | 1 |
| Current exercise intentions | |  |  |  |  |  |  |  |  |  |  |  |  |  |  |  |  |  |  |  |  |  |  |  |  |  |  |  |  |
|  | Frequency (how often the user wants to exercise/week) |  |  |  |  |  |  |  |  |  |  |  |  |  |  |  |  | X |  |  |  |  |  |  |  |  |  |  | 1 |
|  | Intensity (intensity the user wants the exercise to be) |  |  |  |  |  |  |  |  |  |  |  |  |  |  |  |  |  |  |  |  |  | X |  |  |  |  |  | 1 |
|  | Time (total exercise minutes/week or total minutes/session) |  |  |  |  |  |  |  |  |  |  |  |  |  |  |  |  |  |  |  |  |  | X |  |  |  |  |  | 1 |
|  | Type (types of exercise the user wants to engage in) |  |  |  |  |  |  |  |  |  |  |  |  |  |  |  |  |  |  |  |  |  | X |  | X |  |  |  | 2 |
|  | Goal for using the app (get active / get fit / stay fit / get strong / manage weight) |  |  |  |  |  |  |  |  |  |  |  |  |  |  |  |  | X | X |  |  |  |  |  |  |  |  |  | 2 |
| User update on re-open or re-login | |  |  |  |  |  |  |  |  |  |  |  |  |  |  |  |  |  |  |  |  |  |  |  |  |  |  |  |  |
|  | Personal details (height, weight, etc) |  |  |  |  | X |  |  |  |  |  |  |  |  |  |  |  |  |  |  |  |  | X |  |  |  |  |  | 1 |
|  | Exercise behaviours (e.g., doing 2 sessions a week instead of 4) |  |  |  |  | X |  |  |  |  |  |  |  |  |  |  |  |  |  |  |  |  |  |  |  |  |  |  | 1 |
|  | Available equipment (small set of dumbbells, resistance band, etc) |  |  |  |  | X |  |  |  |  |  |  |  |  |  |  |  |  |  |  |  |  |  |  |  |  |  |  | 1 |
|  | Exercise intentions (e.g., no longer want strength training) |  |  |  |  |  |  |  |  |  |  |  |  |  |  |  |  |  |  |  |  |  | X |  |  |  |  |  | 1 |
