# Supplementary material for: Evaluating Evidence-Based Content, Features of Exercise Instruction, and Expert Involvement in Physical Activity Apps for Pregnant Women: Systematic Search and Content Analysis
Source: JMIR Mhealth Uhealth. 2022 Jan 19;10(1):e31607. doi: 10.2196/31607 (PMC8811692; doi:10.2196/31607)
Supplement: Multimedia Appendix 5 [file mhealth_v10i1e31607_app5.docx]

**Multimedia Appendix 5. Frequency, intensity, time (total time accumulated throughout the week), and type principle of exercise and progression.**

*Information on the recommended frequency, intensity, time and types (FITT principles) exercise during pregnancy (detailed).*

| App Identifier | | 01 | 02 | 03 | 04 | 05 | 06 | 07 | 08 | 09 | 10 | 11 | 12 | 13 | 14 | 15 | 16 | 17 | 18 | 19 | 20 | 21 | 22 | 23 | 24 | 25 | 26 | 27 | n |
| --- | --- | --- | --- | --- | --- | --- | --- | --- | --- | --- | --- | --- | --- | --- | --- | --- | --- | --- | --- | --- | --- | --- | --- | --- | --- | --- | --- | --- | --- |
| Frequency of exercise | |  |  |  |  |  |  |  |  |  |  |  |  |  |  |  |  |  |  |  |  |  |  |  |  |  |  |  |  |
|  | Exercise on most, if not all days of the week | X | X | X | X |  | X | X | X | X | X |  | X |  |  |  |  |  |  | X | X |  |  |  |  | X |  |  | 13 |
|  | 2 sessions of resistance-based exercise per week |  |  |  |  |  |  | X |  | X |  |  |  |  |  |  |  |  |  |  | X |  |  |  |  |  |  |  | 3 |
| Intensity of exercise | |  |  |  |  |  |  |  |  |  |  |  |  |  |  |  |  |  |  |  |  |  |  |  |  |  |  |  |  |
|  | Light intensity | X | X | X |  |  |  |  |  |  | X |  |  |  | X |  | X | X | X |  |  |  |  |  |  | X |  | X | 10 |
|  | Moderate intensity | X |  | X |  |  |  | X |  | X | X |  |  |  | X |  | X | X |  | X | X |  |  |  |  |  |  |  | 10 |
|  | Vigorous intensity |  |  | X |  |  |  |  |  | X |  |  |  |  |  |  |  | X |  | X |  |  |  |  |  |  |  |  | 4 |
| Indication of Intensity | |  |  |  |  |  |  |  |  |  |  |  |  |  |  |  |  |  |  |  |  |  |  |  |  |  |  |  |  |
|  | Use Talk Test to judge intensity of exercise |  |  |  |  |  |  |  |  | X | X |  |  |  |  |  |  |  | X |  | X | X |  |  |  |  |  |  | 5 |
|  | Use Borg Rating of Perceived Exertion (RPE) Scale |  |  |  |  |  |  |  |  | X | X |  |  |  |  |  |  |  | X |  | X |  |  |  |  |  |  |  | 4 |
|  | Use Heart Rate zones (based on age & fitness level) |  |  |  |  |  |  | X |  | X |  |  |  |  |  |  |  |  |  |  | X |  |  |  |  |  |  |  | 3 |
| Time (or duration) of exercise | |  |  |  |  |  |  |  |  |  |  |  |  |  |  |  |  |  |  |  |  |  |  |  |  |  |  |  |  |
|  | Exercise for 30 minutes per day | X |  | X |  |  |  |  |  | X | X |  |  |  |  |  | X |  |  |  |  | X |  |  |  |  |  |  | 6 |
|  | Accumulate at least 150 minutes of exercise/week |  |  | X |  |  |  |  |  | X | X |  |  |  | X |  |  |  |  |  | X |  |  |  |  |  |  |  | 5 |
|  | Exercise for at least 15 minutes/session |  | X |  |  |  |  |  |  | X |  |  |  |  |  |  | X |  |  |  | X |  |  |  |  |  |  |  | 4 |
|  | Break up exercise into small bouts |  |  | X |  |  |  |  |  | X | X |  |  |  |  |  |  |  |  |  |  |  |  |  |  |  |  |  | 3 |
| Type of exercise | |  |  |  |  |  |  |  |  |  |  |  |  |  |  |  |  |  |  |  |  |  |  |  |  |  |  |  |  |
|  | Yoga | X | X | X | X |  |  | X | X | X | X | X | X |  | X | X | X | X | X |  | X |  | X | X | X |  | X | X | 21 |
|  | Stretching / Flexibility | X |  | X |  | X |  |  |  | X | X |  | X | X |  | X | X | X | X | X | X | X | X |  | X | X | X | X | 19 |
|  | Pelvic floor / Kegel exercises |  | X |  | X |  | X | X | X |  | X |  | X | X | X | X | X | X | X | X | X | X | X |  |  | X |  | X | 19 |
|  | Strength training (resistance or weight) |  |  | X |  | X |  | X |  |  | X |  | X | X | X |  | X | X | X | X | X | X |  |  | X |  |  |  | 14 |
|  | Pilates |  |  | X |  | X |  | X | X |  |  |  |  |  | X | X | X |  |  | X | X |  |  |  | X | X |  |  | 11 |
|  | Walking / Jogging / Running |  | X | X |  | X |  | X |  |  | X |  |  | X | X |  | X | X |  |  |  | X |  |  | X |  |  |  | 11 |
|  | Swimming |  |  | X |  |  |  |  |  |  | X |  |  | X |  |  | X |  |  |  |  | X |  |  |  |  |  |  | 5 |
|  | High Intensity Interval Training (HIIT) |  |  | X |  | X |  | X |  |  |  |  |  |  |  |  |  |  |  |  | X |  |  |  | X |  |  |  | 5 |
|  | Cycling |  |  | X |  |  |  |  |  |  |  |  |  | X |  |  |  |  |  |  |  |  |  |  | X |  |  |  | 3 |
|  | Other |  |  |  |  |  |  |  |  |  |  |  |  |  |  |  |  |  |  |  |  |  |  |  |  |  |  |  |  |
|  | Pranayama (deep breathing exercises) |  | X |  |  |  |  | X |  | X |  |  |  |  |  |  | X | X |  |  |  |  |  |  |  |  |  | X | 6 |
|  | Barre / Dance |  |  |  |  |  |  | X |  |  |  |  |  |  |  |  | X |  |  |  | X | X | X |  |  |  |  |  | 5 |
|  | Kickboxing |  |  |  |  |  |  | X |  |  |  |  |  |  |  |  |  |  |  |  |  |  | X |  |  |  |  |  | 2 |
|  | Exercise / Gymnastics ball |  |  |  |  |  |  |  | X |  |  |  |  |  |  |  | X |  |  |  |  |  |  |  |  |  |  |  | 2 |

*Information on the progression of exercise during pregnancy (detailed).*

| App Identifier | | 01 | 02 | 03 | 04 | 05 | 06 | 07 | 08 | 09 | 10 | 11 | 12 | 13 | 14 | 15 | 16 | 17 | 18 | 19 | 20 | 21 | 22 | 23 | 24 | 25 | 26 | 27 | n |
| --- | --- | --- | --- | --- | --- | --- | --- | --- | --- | --- | --- | --- | --- | --- | --- | --- | --- | --- | --- | --- | --- | --- | --- | --- | --- | --- | --- | --- | --- |
| Progression of exercise during pregnancy | |  |  |  |  |  |  |  |  |  |  |  |  |  |  |  |  |  |  |  |  |  |  |  |  |  |  |  |  |
|  | If sedentary or inactive, commence exercise slowly & progress toward the recommended guidelines | X |  | X |  |  |  | X | X | X | X |  |  |  | X |  | X |  |  |  | X |  |  |  |  |  |  |  | 9 |
|  | As pregnancy progresses, anatomical & physiological / metabolic changes mean that modifications to some exercises are required |  |  |  |  |  |  | X | X | X | X |  |  |  | X |  | X |  | X |  | X |  |  |  |  |  |  |  | 8 |
|  | Women who are considering high volumes of exercise training should seek advice & guidance from a health professional who is knowledgeable about the effects of high-level training on maternal and fetal outcomes |  |  |  |  |  |  |  | X |  | X |  |  |  |  |  | X |  |  |  |  |  |  |  |  |  |  |  | 3 |
|  | If healthy & already active, you do not need to seek medical clearance for exercise during pregnancy |  |  | X |  |  |  |  |  | X |  |  |  |  |  |  |  |  |  |  |  |  |  |  |  |  |  |  | 2 |
|  | Break up long periods of sitting as often as possible | X |  |  |  |  |  |  |  |  |  |  |  |  |  |  | X |  |  |  |  |  |  |  |  |  |  |  | 2 |
