# Supplementary material for: Evaluating Evidence-Based Content, Features of Exercise Instruction, and Expert Involvement in Physical Activity Apps for Pregnant Women: Systematic Search and Content Analysis
Source: JMIR Mhealth Uhealth. 2022 Jan 19;10(1):e31607. doi: 10.2196/31607 (PMC8811692; doi:10.2196/31607)
Supplement: Multimedia Appendix 6 [file mhealth_v10i1e31607_app6.docx]

**Multimedia Appendix 6. Safety, benefits, and considerations.**

*Information about safe exercises and activities to avoid (detailed).*

| App Identifier | | 01 | 02 | 03 | 04 | 05 | 06 | 07 | 08 | 09 | 10 | 11 | 12 | 13 | 14 | 15 | 16 | 17 | 18 | 19 | 20 | 21 | 22 | 23 | 24 | 25 | 26 | 27 | n |
| --- | --- | --- | --- | --- | --- | --- | --- | --- | --- | --- | --- | --- | --- | --- | --- | --- | --- | --- | --- | --- | --- | --- | --- | --- | --- | --- | --- | --- | --- |
| Exercises considered safe | |  |  |  |  |  |  |  |  |  |  |  |  |  |  |  |  |  |  |  |  |  |  |  |  |  |  |  |  |
|  | Pelvic floor muscle exercises |  | X |  | X |  | X | X | X | X | X |  |  |  | X | X | X |  | X |  | X | X |  |  |  | X |  | X | 15 |
|  | Aerobic exercise (walking / cycling / swimming) | X | X | X |  |  |  | X | X | X | X |  |  | X | X |  | X |  | X |  | X | X |  |  |  |  |  |  | 13 |
|  | Muscle strengthening exercises (using body weight, weights or resistance bands) |  |  | X |  |  |  | X | X | X | X |  |  | X | X | X | X |  | X |  | X | X |  |  |  |  |  |  | 12 |
|  | Pregnancy-specific classes |  |  |  |  |  |  | X |  |  | X |  |  |  | X |  | X |  |  |  | X | X |  |  | X |  |  | X | 8 |
|  | Other |  |  |  |  |  |  |  |  |  |  |  |  |  |  |  |  |  |  |  |  |  |  |  |  |  |  |  |  |
|  | Yoga / Stretching / Pilates |  | X | X | X |  |  |  |  | X | X |  |  |  |  | X | X |  |  |  |  |  |  |  |  |  |  | X | 8 |
|  | Exercise ball / Gym machines (non-specified) |  |  | X |  |  |  |  |  |  | X |  |  |  |  |  | X |  |  |  |  |  |  |  |  |  |  |  | 3 |
|  | Pranayama (deep breathing exercises) |  |  | X |  |  |  |  |  |  | X |  |  |  |  |  |  |  |  |  |  |  |  |  |  |  |  |  | 2 |
|  | Trekking / Hiking |  |  | X |  |  |  |  |  |  | X |  |  |  |  |  |  |  |  |  |  |  |  |  |  |  |  |  | 2 |
|  | High Intensity Interval Training (HIIT) |  |  | X |  |  |  |  |  |  |  |  |  |  |  |  |  |  |  |  |  |  |  |  |  |  |  |  | 1 |
|  | Impact sports (basketball / racquet sport) |  |  | X |  |  |  |  |  |  |  |  |  |  |  |  |  |  |  |  |  |  |  |  |  |  |  |  | 1 |
| Exercises considered unsafe | |  |  |  |  |  |  |  |  |  |  |  |  |  |  |  |  |  |  |  |  |  |  |  |  |  |  |  |  |
|  | Risk of falling (activity requiring balance, coordination & agility) |  | X | X |  |  |  |  | X | X | X |  |  |  | X | X | X |  | X |  | X | X |  |  |  |  |  | X | 12 |
|  | Risk of contact / collision (e.g., basketball / soccer) |  |  | X |  |  |  |  | X | X | X |  |  |  | X |  | X |  | X |  | X | X |  |  |  |  |  |  | 9 |
|  | Long periods of laying in the supine position |  |  |  |  |  |  |  | X | X | X |  |  | X | X |  | X |  | X |  | X | X |  |  |  |  |  |  | 9 |
|  | Heavy lifting (weights / lifting weight overhead) |  |  |  |  |  |  |  | X |  | X |  |  |  | X |  | X |  | X |  | X |  |  |  |  |  |  |  | 6 |
|  | Significant changes in pressure (sky diving / scuba diving) |  |  |  |  |  |  |  |  | X | X |  |  |  | X |  |  |  |  |  | X | X |  |  |  |  |  |  | 5 |
|  | Exercise at high altitude |  |  |  |  |  |  |  | X |  | X |  |  |  |  |  |  |  |  |  |  |  |  |  |  |  |  |  | 2 |
|  | Long periods of standing still |  |  |  |  |  |  |  |  |  | X |  |  |  |  |  |  |  |  |  |  |  |  |  |  |  |  |  | 1 |
|  | Other |  |  |  |  |  |  |  |  |  |  |  |  |  |  |  |  |  |  |  |  |  |  |  |  |  |  |  |  |
|  | Excessive twisting / backbends / contortion | X |  |  |  |  |  |  | X | X | X |  |  |  | X | X | X |  |  |  |  |  |  |  |  |  |  | X | 8 |
|  | Skiing / skating |  |  |  |  |  |  |  |  | X | X |  |  |  | X |  |  |  |  |  |  | X |  |  |  |  |  |  | 4 |
|  | Horseback riding / bouncing / jarring / jumping |  |  |  |  |  |  |  |  | X | X |  |  |  | X |  | X |  |  |  |  |  |  |  |  |  |  |  | 4 |
|  | Advanced abdominal moves (sit ups / crunches) |  |  |  |  |  |  |  |  |  | X |  |  |  |  |  | X |  |  |  |  |  |  |  |  |  |  | X | 3 |
|  | Laying on stomach | X |  |  |  |  |  |  | X |  |  |  |  |  |  |  |  |  |  |  |  |  |  |  |  |  |  | X | 3 |
|  | Holding breath or forcing breath | X |  |  |  |  |  |  |  |  | X |  |  |  |  |  |  |  |  |  |  |  |  |  |  |  |  | X | 3 |
|  | Running / Cycling |  |  |  |  |  |  |  | X |  | X |  |  |  |  |  | X |  |  |  |  |  |  |  |  |  |  |  | 3 |
|  | Bungee jumping |  |  |  |  |  |  |  |  |  | X |  |  |  |  |  |  |  |  |  |  |  |  |  |  |  |  |  | 1 |

*Additional considerations relating to exercise during pregnancy (detailed).*

| App Identifier | | 01 | 02 | 03 | 04 | 05 | 06 | 07 | 08 | 09 | 10 | 11 | 12 | 13 | 14 | 15 | 16 | 17 | 18 | 19 | 20 | 21 | 22 | 23 | 24 | 25 | 26 | 27 | *n* |
| --- | --- | --- | --- | --- | --- | --- | --- | --- | --- | --- | --- | --- | --- | --- | --- | --- | --- | --- | --- | --- | --- | --- | --- | --- | --- | --- | --- | --- | --- |
| Additional considerations | |  |  |  |  |  |  |  |  |  |  |  |  |  |  |  |  |  |  |  |  |  |  |  |  |  |  |  |  |
|  | Stay well hydrated | X |  | X |  |  | X | X | X | X | X |  |  |  | X |  | X |  | X | X | X | X | X |  | X |  |  |  | 15 |
|  | Always wear appropriate clothing (appropriate shoes, non-restrictive clothing & supportive bra) |  | X | X |  |  |  |  | X | X | X |  |  | X | X |  | X |  |  |  |  | X |  |  |  |  |  |  | 9 |
|  | Adjust exercise in excessively hot weather or high humidity |  |  |  |  |  |  |  |  |  | X |  |  |  | X |  | X |  |  |  | X | X |  |  |  |  | X |  | 6 |
|  | Try to ensure energy intake is in line with recommended gestational weight gain |  |  |  |  |  |  | X | X |  | X |  |  |  |  |  |  |  |  |  |  |  |  |  |  |  |  |  | 3 |
|  | Minimise amount of time spent in prolonged sitting |  |  |  |  |  |  |  |  |  |  |  |  |  |  |  | X |  |  |  |  |  |  |  |  |  |  |  | 1 |
|  | Other |  |  |  |  |  |  |  |  |  |  |  |  |  |  |  |  |  |  |  |  |  |  |  |  |  |  |  |  |
|  | Remember to warm up prior to exercise | X |  |  |  |  |  |  |  |  |  |  |  |  | X |  |  |  |  |  | X | X |  |  |  |  |  |  | 4 |
|  | Do not unnecessarily strain yourself |  | X |  |  |  |  |  |  |  |  |  |  |  |  |  |  |  |  |  | X |  |  |  |  |  |  |  | 2 |
|  | Listen to your body |  |  |  |  |  |  |  |  | X |  |  |  |  |  |  |  |  |  |  | X |  |  |  |  |  |  |  | 2 |
|  | Skip exercise on days that you do not feel up to exercising |  |  |  |  |  |  |  |  |  | X |  |  |  | X |  |  |  |  |  |  |  |  |  |  |  |  |  | 2 |
|  | Avoid sitting on a hard surface for too long | X |  |  |  |  |  |  |  |  |  |  |  |  |  |  |  |  |  |  |  |  |  |  |  |  |  |  | 1 |

*Warning signs or symptoms to stop exercise during pregnancy (detailed).*

| App Identifier | | 01 | 02 | 03 | 04 | 05 | 06 | 07 | 08 | 09 | 10 | 11 | 12 | 13 | 14 | 15 | 16 | 17 | 18 | 19 | 20 | 21 | 22 | 23 | 24 | 25 | 26 | 27 | n |
| --- | --- | --- | --- | --- | --- | --- | --- | --- | --- | --- | --- | --- | --- | --- | --- | --- | --- | --- | --- | --- | --- | --- | --- | --- | --- | --- | --- | --- | --- |
| Signs or symptoms to cease exercise | |  |  |  |  |  |  |  |  |  |  |  |  |  |  |  |  |  |  |  |  |  |  |  |  |  |  |  |  |
|  | Persistent dizziness / feeling faint – that does not resolve with rest |  |  | X |  | X |  |  |  | X | X |  | X | X | X |  | X |  | X |  | X | X | X |  |  |  |  |  | 12 |
|  | Persistent excessive shortness of breath – that does not resolve with rest |  |  |  |  | X | X |  |  | X | X |  | X | X | X |  |  |  | X |  | X | X | X |  |  |  |  |  | 11 |
|  | Regular painful uterine contractions |  |  |  |  |  |  |  | X | X | X |  | X | X | X |  | X |  | X |  | X |  |  |  |  |  |  |  | 9 |
|  | Vaginal bleeding |  |  |  |  |  |  |  |  | X | X |  | X | X | X |  | X |  | X |  | X |  |  |  |  |  |  |  | 8 |
|  | Heat stress / hyperthermia in 1st trimester |  |  | X |  |  |  |  | X |  | X |  |  | X | X |  | X |  |  |  | X |  |  |  |  |  | X |  | 8 |
|  | Chest pain |  |  | X |  |  |  |  |  | X |  |  | X | X | X |  |  |  | X |  | X |  |  |  |  |  |  |  | 7 |
|  | Persistent loss of fluid from the vagina – indicating possible ruptured membrane |  |  |  |  |  |  |  |  | X |  |  | X | X | X |  | X |  | X |  | X |  |  |  |  |  |  |  | 7 |
|  | Inadequate nutrition |  |  | X |  |  |  | X |  |  |  |  |  |  | X |  | X |  |  |  | X |  |  |  | X |  |  | X | 7 |
|  | Dehydration |  |  | X |  |  |  | X | X |  | X |  |  |  | X |  |  |  |  |  | X |  |  |  |  |  |  |  | 6 |
|  | Severe headache |  |  |  |  |  |  |  |  | X |  |  | X | X |  |  |  |  | X |  | X |  |  |  |  |  |  |  | 5 |
|  | Other |  |  |  |  |  |  |  |  |  |  |  |  |  |  |  |  |  |  |  |  |  |  |  |  |  |  |  |  |
|  | Severe discomfort / Pain |  |  | X |  | X |  | X |  |  | X |  | X |  | X |  |  |  | X |  |  |  | X |  |  |  |  | X | 9 |
|  | Nausea / Feeling unwell |  |  |  |  | X |  |  |  |  |  |  |  |  | X |  |  |  | X |  |  | X |  |  |  |  |  | X | 5 |
|  | Muscle weakness |  |  |  |  |  |  |  |  | X |  |  | X | X |  |  |  |  | X |  |  |  |  |  |  |  |  |  | 4 |
|  | Absence of / decreased fetal movement |  |  |  |  |  |  |  |  | X | X |  | X |  |  |  |  |  | X |  |  |  |  |  |  |  |  |  | 4 |
|  | Contractions becoming more intense / frequent |  |  |  |  |  |  |  |  | X | X |  | X |  |  |  |  |  | X |  |  |  |  |  |  |  |  |  | 4 |
|  | Heart palpitations or irregular heartbeat |  |  |  |  |  |  |  |  |  |  |  | X | X | X |  |  |  | X |  |  |  |  |  |  |  |  |  | 4 |
|  | Feelings of exhaustion or fatigue |  |  |  |  |  |  |  |  |  |  |  | X |  | X |  | X |  |  |  |  |  |  |  |  |  |  |  | 3 |
|  | Pain or swelling in lower leg |  |  |  |  |  |  |  |  |  |  |  | X | X |  |  |  |  | X |  |  |  |  |  |  |  |  |  | 3 |
|  | Burning or difficulty in urinating |  |  |  |  |  |  | X |  |  |  |  |  |  |  |  |  |  | X |  |  |  |  |  |  |  |  |  | 2 |
|  | Blurred vision or dizziness |  |  | X |  |  |  |  |  |  |  |  |  |  |  |  | X |  |  |  |  |  |  |  |  |  |  |  | 2 |
