# Supplementary material for: Evaluating Evidence-Based Content, Features of Exercise Instruction, and Expert Involvement in Physical Activity Apps for Pregnant Women: Systematic Search and Content Analysis
Source: JMIR Mhealth Uhealth. 2022 Jan 19;10(1):e31607. doi: 10.2196/31607 (PMC8811692; doi:10.2196/31607)
Supplement: Multimedia Appendix 7 [file mhealth_v10i1e31607_app7.docx]

**Multimedia Appendix 7. Delivery, format, and features of exercise instruction.**

*Features used to present and instruct exercise, information to help inform selection of exercise, and options to modify exercise (detailed).*

| App Identifier | | 01 | 02 | 03 | 04 | 05 | 06 | 07 | 08 | 09 | 10 | 11 | 12 | 13 | 14 | 15 | 16 | 17 | 18 | 19 | 20 | 21 | 22 | 23 | 24 | 25 | 26 | 27 | n |
| --- | --- | --- | --- | --- | --- | --- | --- | --- | --- | --- | --- | --- | --- | --- | --- | --- | --- | --- | --- | --- | --- | --- | --- | --- | --- | --- | --- | --- | --- |
| Features used to present exercise | |  |  |  |  |  |  |  |  |  |  |  |  |  |  |  |  |  |  |  |  |  |  |  |  |  |  |  |  |
|  | A series of individual exercises | X | X | X |  |  | X |  |  | X | X | X |  | X | X | X | X |  |  | X | X | X |  | X |  | X | X | X | 18 |
|  | A series of individual workouts  (contains a series of pre-determined exercises) |  |  |  | X | X |  | X | X |  |  |  | X |  |  |  |  |  | X | X | X |  | X |  | X | X | X |  | 12 |
|  | A series of exercise programs  (contains a series of pre-determined workouts) |  |  |  |  |  |  |  |  |  |  |  |  |  |  |  |  | X | X |  |  |  | X |  | X |  | X |  | 5 |
| Features used to instruct exercise | |  |  |  |  |  |  |  |  |  |  |  |  |  |  |  |  |  |  |  |  |  |  |  |  |  |  |  |  |
|  | Written cues instructing exercise | X | X | X |  |  | X |  | X | X | X | X | X | X | X | X | X | X |  | X |  | X | X | X | X | X | X | X | 22 |
|  | Video/s demonstrating exercise |  | X |  | X | X |  | X |  | X |  |  | X |  |  |  | X | X | X | X | X |  | X | X | X | X |  | X | 16 |
|  | Spoken cues instructing exercise |  |  |  | X | X |  | X |  | X |  |  | X |  |  | X |  | X | X | X | X |  | X | X | X |  |  | X | 14 |
|  | Still images / pictures demonstrating exercise |  | X |  |  |  | X |  |  |  |  | X | X | X | X | X | X | X |  | X | X |  |  |  |  |  | X |  | 12 |
| Information to inform selection of exercise | |  |  |  |  |  |  |  |  |  |  |  |  |  |  |  |  |  |  |  |  |  |  |  |  |  |  |  |  |
|  | Duration of exercise session (e.g., 20 minutes) |  |  | X | X | X | X | X |  |  | X |  | X |  |  |  | X | X | X | X | X |  | X | X | X | X |  | X | 17 |
|  | Type of exercise session (e.g., targets lower body) | X |  |  |  | X | X | X |  |  |  |  |  |  |  |  | X | X | X |  | X |  | X | X | X | X |  | X | 13 |
|  | Trimester (e.g., 2^nd^ trimester) |  |  |  |  | X |  | X |  |  |  |  |  |  |  |  | X | X | X | X | X |  | X | X | X |  |  | X | 11 |
|  | Equipment required (e.g., a small set of dumbbells) |  |  |  |  |  |  | X |  |  |  |  |  |  |  |  | X | X | X | X | X |  | X | X | X |  |  | X | 10 |
|  | Frequency of exercise (e.g., 3 rounds, 10 repetitions per round) |  |  |  | X | X | X |  |  |  | X |  | X |  |  |  | X |  |  |  | X |  |  |  | X |  |  |  | 8 |
|  | Intensity of exercise session (e.g., short bouts of high intensity exercise) |  |  |  |  |  | X |  |  |  | X |  |  |  |  |  | X | X | X |  | X |  | X |  |  |  |  |  | 7 |
|  | Experience level required (e.g., beginners) |  |  |  |  |  | X |  |  |  |  |  |  |  |  |  |  | X |  |  | X |  | X |  |  |  |  |  | 4 |
| Allowance of user modification | |  |  |  |  |  |  |  |  |  |  |  |  |  |  |  |  |  |  |  |  |  |  |  |  |  |  |  |  |
|  | Duration of exercise |  |  |  | X |  |  |  |  |  |  |  |  |  |  |  |  | X |  |  |  |  | X |  |  |  |  |  | 3 |
|  | Frequency of exercise |  |  |  | X |  |  |  |  |  |  |  |  |  |  |  |  |  |  |  |  |  | X |  |  |  |  |  | 2 |
|  | Intensity of exercise |  |  |  |  |  |  |  |  |  |  |  |  |  |  |  |  | X |  |  |  |  | X |  |  |  |  |  | 2 |
|  | Type of exercise |  |  |  |  |  |  |  |  |  |  |  |  |  |  |  |  | X |  |  |  |  | X |  |  |  |  |  | 2 |

*Developer recognised credentials, experiential credibility, and referenced sources of information (detailed).*

| App Identifier | | 01 | 02 | 03 | 04 | 05 | 06 | 07 | 08 | 09 | 10 | 11 | 12 | 13 | 14 | 15 | 16 | 17 | 18 | 19 | 20 | 21 | 22 | 23 | 24 | 25 | 26 | 27 | n |
| --- | --- | --- | --- | --- | --- | --- | --- | --- | --- | --- | --- | --- | --- | --- | --- | --- | --- | --- | --- | --- | --- | --- | --- | --- | --- | --- | --- | --- | --- |
| Developer credentials and credibility | |  |  |  |  |  |  |  |  |  |  |  |  |  |  |  |  |  |  |  |  |  |  |  |  |  |  |  |  |
|  | Implies developer credibility or experience |  |  |  |  | X |  | X |  |  |  |  |  |  |  |  |  | X | X | X | X |  |  |  |  | X | X |  | 8 |
|  | Specifies developer formal qualification/s |  |  |  |  |  |  | X |  | X |  |  |  |  |  |  |  | X | X |  |  |  |  |  |  | X |  | X | 6 |
| Reference to recognised sources of information | |  |  |  |  |  |  |  |  |  |  |  |  |  |  |  |  |  |  |  |  |  |  |  |  |  |  |  |  |
|  | Government guidelines (exercise guidelines that incorporate pregnancy &/or exercise during pregnancy guidelines) |  | X |  | X |  |  | X |  | X |  |  |  |  | X |  |  |  |  |  | X | X |  |  |  |  |  |  | 7 |
|  | Academic literature |  |  |  | X |  |  | X |  | X | X |  |  |  |  |  |  |  |  |  | X |  |  |  |  |  |  | X | 6 |
|  | Obstetrics-related guidelines |  |  |  | X |  |  |  |  | X | X |  |  |  |  |  |  |  |  |  | X |  |  |  |  |  |  | X | 5 |
